# Single-cell Bayesian deconvolution

**DOI:** 10.1016/j.isci.2023.107941

**Published:** 2023-09-19

**Authors:** Gabriel Torregrosa-Cortés, David Oriola, Vikas Trivedi, Jordi Garcia-Ojalvo

**Affiliations:** 1 Department of Medicine and Life Sciences, Universitat Pompeu Fabra, Barcelona Biomedical Research Park, 08003 Barcelona, Spain; 2 Department of Physics, Universitat Politècnica de Catalunya, 08028 Barcelona, Spain; 3 EMBL Barcelona, Barcelona Biomedical Research Park, 08003 Barcelona, Spain; 4 EMBL Heidelberg, Developmental Biology Unit, 69117 Heidelberg, Germany

**Keywords:** Technical aspects of cell biology, Biocomputational method, Complex system biology, Optical Signal Processing

## Abstract

Individual cells exhibit substantial heterogeneity in protein abundance and activity, which is frequently reflected in broad distributions of fluorescently labeled reporters. Since all cellular components are intrinsically fluorescent to some extent, the observed distributions contain background noise that masks the natural heterogeneity of cellular populations. This limits our ability to characterize cell-fate decision processes that are key for development, immune response, tissue homeostasis, and many other biological functions. It is therefore important to separate the contributions from signal and noise in single-cell measurements. Addressing this issue rigorously requires deconvolving the noise distribution from the signal, but approaches in that direction are still limited. Here, we present a non-parametric Bayesian formalism that performs such a deconvolution efficiently on multidimensional measurements, providing unbiased estimates of the resulting confidence intervals. We use this approach to study the expression of the mesodermal transcription factor Brachyury in mouse embryonic stem cells undergoing differentiation.

## Highlights

• A Bayesian method robustly removes autofluorescence from flow cytometry data
• The method provides unbiased error estimates and natural clustering of the data
• The code is publicly available as an efficient Julia package

## Graphical abstract

**Figure:**
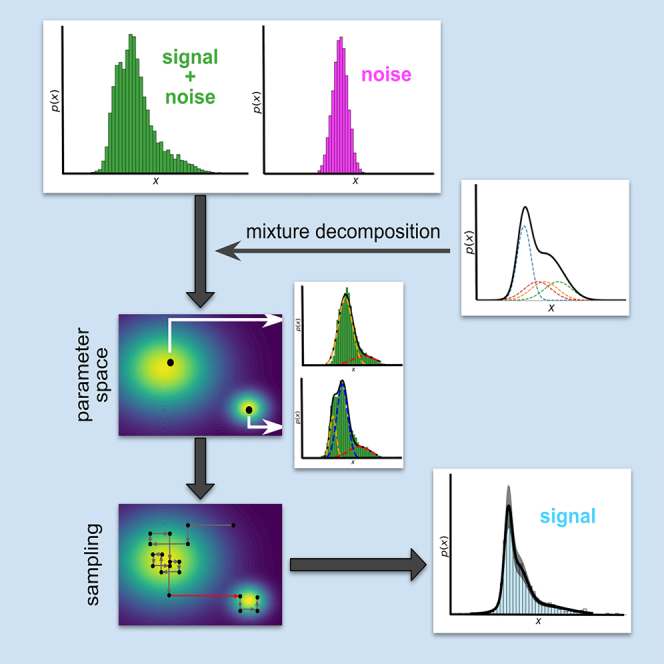

## Introduction

The inherent stochasticity of biological processes leads to substantial heterogeneity even among genetically identical cells in the same environment.^1^^,^^2^^,^^3^ The degree to which this heterogeneity affects, or even dictates, cellular decision-making in most situations is still an open question. This issue is of paramount importance in processes such as mammalian development, where hundreds (if not thousands^4^) of distinct cell types (cell states) emerge from a small number of identical undifferentiated cells.^5^^,^^6^^,^^7^ Identifying the molecular mechanisms underlying these cell-fate decision programs and their interplay with cellular heterogeneity^8^^,^^9^ requires a rigorous quantification of cellular states across large numbers of cells.

Flow cytometry enables monitoring the distributions of abundances and activities of selected proteins for thousands of cells at a time, using fluorescently labeled markers. Cells, however, have a non-negligible amount of autofluorescence in the emission spectrum of most fluorescent probes (500–700 nm). In what follows, we indistinctly refer to this autofluorescence as *noise*, since for our purposes it corresponds to an undesired random signal on top of our signal of interest. This backround noise must be subtracted from the total signal emitted by fluorescently labeled cells,^10^ in order to adequately relate the signal distribution provided by the cytometer to the mechanisms regulating the expression and/or activity of the protein of interest. Several standard methods exist for addressing this issue on a cell-by-cell basis. One can use, for instance, additional fluorescence channels outside the emission spectrum of the fluorophores serving as regressor variables.^11^^,^^12^ Other experimental setups have been developed to deal with autofluorescence, such as introducing a second laser system that provides independent measurements of both the total and autofluorescence signals in the same sample,^13^ and even combining *in silico* simulations with experimental measurements of quantum-dot markers to track cellular proliferation.^14^ Beyond issues of cost or accessibility, the effectivity of such solutions is limited, because there is no guarantee that the autofluorescence from another channel, or from another excitation source, is a good proxy for the autofluorescence in our channel of interest.

Commonly, rather than dedicating measurement resources to assess autofluorescence, control measurements of unlabeled cells are used to set a baseline of the signal coming from the naturally present autofluorescent components in the cell. This procedure, however, does not lead to a quantitative determination of the distribution of the signal coming *exclusively* from the fluorescent probe. Such quantitative assessment would require deconvolving the fluorescence distribution obtained in labeled cells from the one produced by unlabeled cells. Here, we propose a non-parametric Bayesian approach to this deconvolution problem, applicable to multichannel measurements. The method is robust and efficient, requiring cell numbers not larger than those typically considered in standard flow cytometry runs, and gives natural confidence intervals of the target distributions, which makes it attractive for a variety of applications.

There is an extensive statistics literature addressing the additive deconvolution problem (We do not discuss in what follows other deconvolution problems that are not relevant to our situation, such as those related with composition of distributions, which usually require complete knowledge of the noise distribution^15^^,^^16^). A common set of deconvolution methods are kernel-based approaches, such as those relying on Fourier transforms,^17^^,^^18^^,^^19^^,^^20^^,^^21^^,^^22^^,^^23^ which use the fact that in Fourier space, a deconvolution is simply the product of two functions. Two problems arise from such methods that limit their applicability in practical cases. First, Fourier transforms (and other methods that use orthogonal local bases, such as wavelets^24^) can have negative values in part of their domain, which is incompatible with the positive-valued requirement that probability densities must obey. Consequently, kernel-based methods lead to deconvolved pseudo-distributions with artificial features, which are hardly interpretable for practical applications. Second, these methods usually lead to point estimates, and therefore do not provide native confidence intervals (i.e., without applying additional statistical approximations) that allow us to assess the quality of the inferred target distribution. Having a confidence interval around the deconvolved distribution is crucial for practical applications. In general, depending on the levels of noise and the size of the dataset, the final convolved distribution could be equally well described by many different target distributions with quantitative and qualitative dissimilarities. A confidence interval introduces an error estimate representing how different are the various compatible solutions given the data and thus how much one should trust the inferred target distribution in each case.

A second class of deconvolution approaches are likelihood-based methods,^25^ which estimate the unknown target distribution using maximum likelihood approaches. As in the case of kernel-based methods, these approaches provide us with point estimates, and usually assume exact knowledge of the noise distribution.^16^ Additionally, these methods commonly use kernel density estimation to represent the data,^10^ which is rather limited when dealing with multidimensional datasets. Finally, a third class of methods involve Bayesian inference,^26^^,^^27^^,^^28^ which does not require complete knowledge of the noise distribution and naturally provides confidence intervals of the estimates obtained. Bayesian approaches have also been applied, in combination with mixture models (described in the following section), to cluster data in flow cytometry analyses.^29^ However, those methods have only been applied so far to repeated measurements of the same individual entities that are being monitored (in our case, cells), which is not a realistic possibility in standard flow cytometry. Our non-parametric Bayesian approach does not require repeated measurements and retains all the aforementioned advantages of Bayesian methods. We have implemented the procedure in a Julia package available in GitHub (https://github.com/dsb-lab/scBayesDeconv.jl).

The work is structured as follows. First, we introduce the mathematical description of the additive convolution problem in the context of flow cytometry data, and explain our proposed deconvolution method based on non-parametric Bayesian models. Second, we validate our method using synthetic datasets with known target distributions, and compare its results to other existing methods. Third, we further test our method in real flow cytometry data of mouse embryonic stem cells undergoing differentiation. We test the dataset in two conditions. Initially, we treat our cells with a low concentration of a fluorescent dye (which masks the real flow cytometry signal and acts as an external noise), and validate our method by deconvolving the noise coming from the dye and comparing it to the control case where the dye was not added. This offers evidence of the robustness of the method in real applications while having a ground truth. Additionally, we perform the deconvolution using several channels from the dataset, to show the effectiveness of the method in multichannel measurements, and comment on the complementarity of our approach to other processing steps common in analysis pipelines of flow cytometry data. We conclude by discussing the limitations of the method and possible ways to improve it in future work.

## Results

### Theoretical definition of the problem

Consider a population of cells containing fluorescent markers that label the abundances or activities (e.g., phosphorylation states) of certain proteins of interest. Flow cytometry measurements provide us with the distribution $p_{c}\left(C\right)$ of total fluorescence signal *C* emitted by each individual cell in the population, as measured by the flow cytometer detectors. These signals have two components: the fluorescence *T* emitted exclusively by the target fluorophores that report on the proteins of interest, and the autofluorescence ξ emitted by cellular components other than our fluorescent labels:

(Equation 1) C=T+ξ

If these two components are independent of one another, the distribution $p_{c}\left(C\right)$ of the measured signal takes the form of a *convolution* of the distributions of *T* and ξ:

(Equation 2) $$\left(p_{T}*p_{\xi }\right)\left(C\right):=\int_{0}^{\infty }p_{T}\left(C-\xi \right)\;p_{\xi }\left(\xi \right)\;d\xi$$

Similarly to $p_{c}$, the distribution $p_{\xi }$ of the autofluorescence ξ can be measured in the flow cytometer by using unlabeled cells that are otherwise identical to the labeled ones. On the other hand, the probability distribution of *T*, $p_{T}$, cannot be measured directly. Our goal is to extract (deconvolve) the distribution $p_{T}$ from the measured distributions $p_{c}$ and $p_{\xi }$, considering that we only have a finite set of samples (cells) of *C* and ξ.

In what follows, we first introduce the way in which we describe the distributions involved in the problem. Next, we define the posterior distribution given by the model and the data, and finally we discuss the methods used to explore the parameter space for the deconvolution problem.

#### Mixture model

The vast majority of real datasets (including those generated in flow cytometry) result from complex combinations of variables that cannot be explained in general with simple distributions. In order to adapt flexibly to such conditions, we use mixtures of probability distributions as our basis set. Any function can be arbitrarily well approximated using an adequate choice of basis functions, provided enough components are included in the mixture. We can describe both the target and the noise distributions by independent mixtures of *K* components:

(Equation 3) $$p\left(x|\varphi \right)=\sum_{\eta =1}^{K}\omega_{\eta }B\left(x|\psi_{\eta }\right)$$

where *x* is multivariate in the case of multichannel measurements. $\omega_{\eta }$ denotes the weight of each base $B\left(x|\psi_{\eta }\right)$ in the mixture, and $\psi_{\eta }$ represents its parameters (the means and covariance matrices in the case of multivariate normal distributions) (Throughout the paper we use greek subindices, in particular η and λ, when summing over basis functions, and roman subindices when summing over samples cells). The sets of $\omega_{\eta }$ and $\psi_{\eta }$ are in turn represented by the vector φ in what follows. Using these basis functions, the distribution of the observed signal *C* can be described as a superposition of all the convolutions between them:

(Equation 4) $$p_{c}\left(c|\varphi_{T},\varphi_{\xi }\right)=\sum_{\eta =1}^{K_{T}}\sum_{\lambda =1}^{K_{\xi }}\omega_{\eta }^{T}\omega_{\lambda }^{\xi }\left(B*B\right)\left(c|\psi_{\eta }^{T},\psi_{\lambda }^{\xi }\right)$$

where $\left(B*B\right)\left(c|\psi_{\eta }^{T},\psi_{\lambda }^{\xi }\right)$ represents the convolution of two basis distributions with parameters $\psi_{\eta }^{T}$ and $\psi_{\lambda }^{\xi }$, respectively, and $K_{T}$ and $K_{\xi }$ denote the number of bases used in each of the two mixtures.

The choice of basis functions to describe our data are crucial. We propose to use multivariate normal distributions to describe the data. Normal distributions with unknown mean and covariance have been shown to be flexible enough to represent datasets with high quality, without requiring many more components than non-normal, skewed basis functions such as gamma distributions. Normal basis functions have the additional advantage that their convolution turns out to be another normal distribution with modified parameters.^30^ This last property is especially interesting for deriving exact analytical results for the Bayesian sampling process.

#### Posterior distribution and likelihood

Our goal is to extract, starting from samples of the distributions of total signal *C* and noise ξ, the parameters of the distribution of the target signal *T*. Defining the problem in terms of probability distributions leads very naturally to work with Bayesian methods. According to Bayes’ rule, the posterior distribution that represents the probability of the parameters given the data are

(Equation 5) $$p\left(\varphi_{T},\varphi_{\xi }|c,\xi \right)\propto \left[p\left(c|\varphi_{T},\varphi_{\xi }\right)p\left(\xi |\varphi_{\xi }\right)\right]\left[p\left(\varphi_{T}\right)p\left(\varphi_{\xi }\right)\right]\text{,}$$

where $c=\left\{c_{i}:i=1,\ldots ,N_{c}\right\}$ and $\xi =\left\{\xi_{i}:i=1,\ldots ,N_{\xi }\right\}$ are the sets of observed samples of the total signal and the noise, respectively, with $N_{c}$ and $N_{\xi }$ representing the number of samples in each case.

The first bracket on the right-hand side of Equation 5 is the likelihood function, which corresponds to the probability of the data given the parameters. Under the assumption of independent and identically distributed (iid) observations, this function is given by

(Equation 6) $$L\left(\varphi_{T},\varphi_{\xi }\right)=\prod_{i=1}^{N_{c}}p_{c}\left(c_{i}|\varphi_{T},\varphi_{\xi }\right)\prod_{j=1}^{N_{\xi }}p_{\xi }\left(\xi_{j}|\varphi_{\xi }\right)$$

As can be seen, the information about the noise parameters is contained in both datasets, while the information about the target parameters only appears in the convolved data.

As shown in Equation 5 previously, the posterior distribution can be estimated by multiplying the likelihood by the prior distribution of parameter values. Since the datasets required for a good deconvolution are large, the impact of the prior distribution should be negligible. Therefore, the posterior landscape is effectively described by Equation 6, and the prior distribution is only present in our approach for formal and computational reasons. In any case, since no prior information exists on the parameters that describe the noise and target mixture components, we impose vague priors over the plausible set of parameters (see STAR Methods "Hyperparameter selection").

#### Sampling

The posterior distribution is a complex multimodal (multi-peaked) object containing all the configurations of the target and noise distributions that are consistent with the observed data. A correct and efficient exploration of this distribution is essential to set bounds on the candidate models that explain the data. The Bayesian model, as stated in Equation 5, has no direct closed form. Sampling from this model as stated would require the introduction of complex sampling algorithms such as Markov Chain Monte Carlo (MCMC)^30^ and nested sampling.^31^ These sampling processes are computationally very expensive and scale linearly with sample size, which in the case of flow cytometry datasets is of the orders of tens to hundreds of thousands samples per dataset.

In order to overcome these shortcomings, we propose two approximations to the posterior distribution. The first one decouples the inference of the noise and target distributions parameters as two independent problems (see STAR Methods "Approximated decomposition of the posterior into two separate problems"). The parameters of the noise distribution can be fitted using well-known mixtures of normal distributions, with the target being described by a convolved mixture model. The second approximation simplifies the sampling of the convolved mixture model to enable sampling using standard Gibbs sampling algorithms (see STAR Methods "Gibbs sampling algorithms"). The combination of both approximations allows the posterior to be sampled using Gibbs sampling, which is an efficient MCMC method of sampling from the posterior.

In addition to these approximations, we offer two alternative Bayesian mixtures, namely finite or infinite normal mixture models.^32^ The latter approach has the added advantage over the former of not requiring fixing the number of basis functions to describe the data. However, for most practical purposes, finite normal mixture models are able to fit the data with high accuracy and in a more efficient manner regarding how the corresponding algorithms work.

#### Analysis pipeline

**Figure 1:**
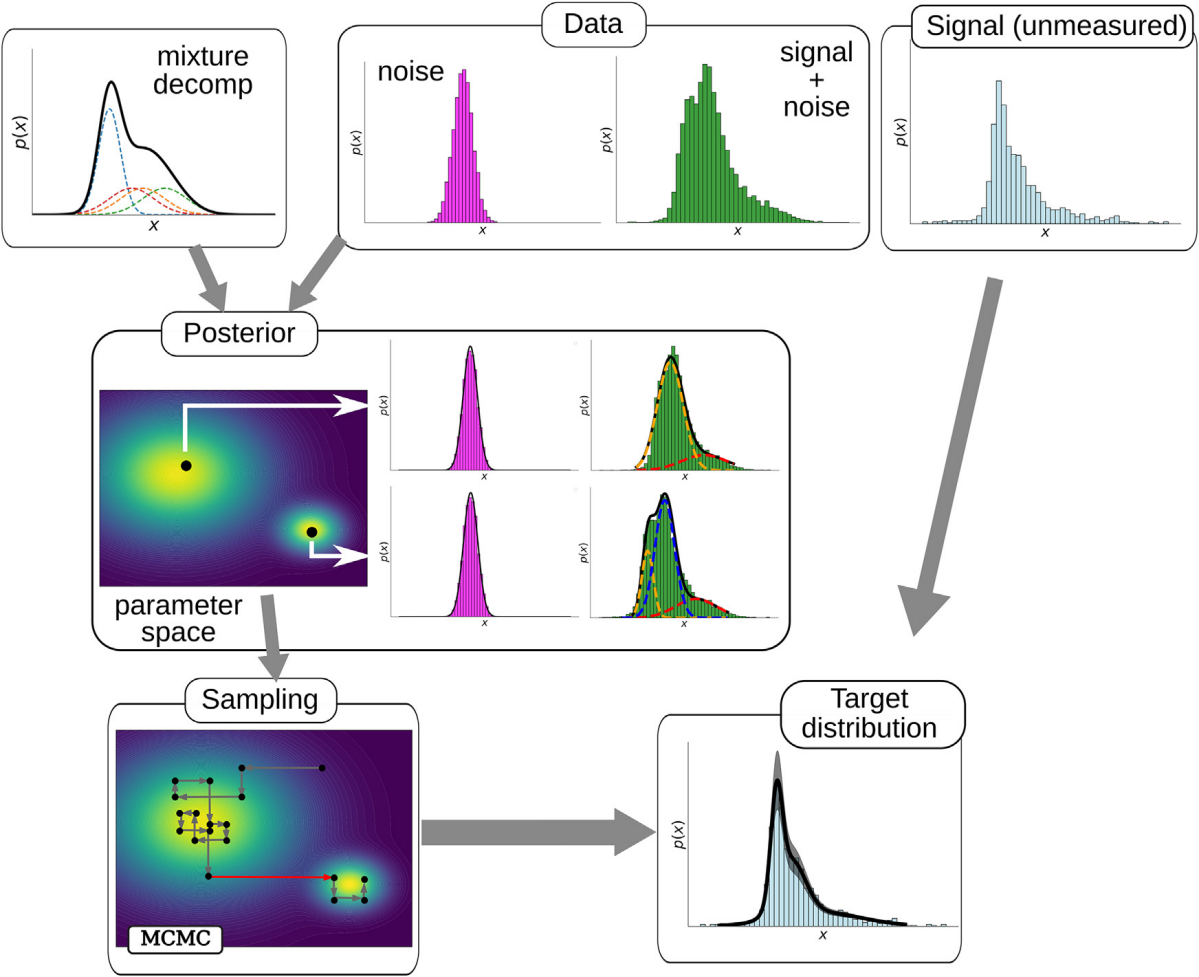
Scheme of the deconvolution process The signal and noise mixture distributions, together with the observed data (top row), define the posterior distribution over the parameter space of the mixture, Equation 6 (middle row). This distribution can present multiple peaks, sometimes degenerate with respect to basis label exchange, each corresponding to a different mixture description of the observed and target distributions. The red arrow in the Gibbs Markov Chain Monte Carlo sampling plot (bottom left) represents an unlikely jump between two peaks separated by a relatively wide probability valley.

Given the concepts and tools described previously, the analysis of the data are performed as shown in Figure 1. First, the distributions of the target and autofluorescence signals are assumed to be described by mixtures (top left panel in Figure 1), as defined by Equations 3 and 4. The data measured experimentally correspond to the autofluorescence signal (noise) and the total signal of the labeled cells (which includes the autofluorescence), as shown in the top right panel of Figure 1. The mixture assumption and the observed data samples allow us to construct the posterior distribution (Equation 5 and middle panel in Figure 1) from the likelihood function defined by Equation 6 and the prior distributions discussed in the STAR Methods "Hyperparameter selection". The posterior distribution is a function of the model parameters (weights of the mixtures and parameters of the basis functions). We next explore (sample) the posterior distribution in parameter space using the Gibbs sampling approximation (bottom left panels in Figure 1). This algorithm provides us with a representative sampling of the parameters of the target distribution, which allows us to compute an average of this distribution and its confidence interval (bottom right panel in the figure).

### Application to synthetic data

**Figure 2:**
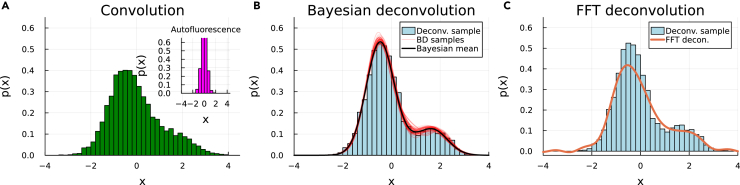
Deconvolution instance for a target bimodal distribution (light blue in B and C) corrupted by a normally distributed noise with a signal-to-noise ratio SNR=2 (magenta distribution in the inset of A), applying the Bayesian Deconvolution method (B) and the FFT method (C) (A and B) The total distribution from which the noise is deconvolved is shown in green in panel (A). The red lines in panel (B) depict samples of the Bayesian fitting process. In this case, the noise distribution is a single normal function with mean $\mu^{\xi }=0$ and standard deviation $\sigma^{\xi }=0.5$, and the target is a mixture of two normal functions with means $\mu_{1}^{T}=-0.43$ and $\mu_{2}^{T}=1.67$, standard deviations $\sigma_{1}^{\xi }=\sigma_{2}^{\xi }=0.6$, and weights $\omega_{1}^{T}=0.8$ and $\omega_{2}^{T}=0.2$.

First, we benchmark the ability of our method to recover the target distribution by using collections of synthetic datasets. In those datasets, the target and noise distributions are known, so we can compare the result of the deconvolution against a ground truth. To do this, we applied our method to the synthetic data described in the STAR Methods "Synthetic data". To prove the robustness of the implementation, we ran the test using four components for both the noise and target distributions. We also avoided checking the full convergence of the algorithm manually. We ran the algorithm in these suboptimal conditions to avoid having to fine-tune the specific parameters, which could lead to positive bias favoring our method in comparison with FFT-based approaches. We contrasted the results of our method with those of a specific FFT approach that does not require knowledge of the autofluorescence distribution.^21^ Figure 2 shows a typical instance of the deconvolution performance of the two methods.

Panel (A) shows in green the total (convolved) signal mimicking the output of a flow cytometry experiment. In this synthetic case, the signal is obtained by forward convolving a target distribution with the characteristics given earlier, shown in light blue in panels (B) and (C), with a noise (auto-fluorescence) distribution, shown in magenta in the inset of panel (A). The goal in this case is to recover (deconvolve) the ground-truth target distribution from the total and noise distributions. Even in the case of the relatively simple target distribution considered here (a bimodal normal mixture), our Bayesian deconvolution method (Figure 2B) performs qualitatively better than the FFT-based method (Figure 2C). Additionally, the FFT method leads to oscillatory components in the deconvolution, and correspondingly to artifactual negative values in the probability distribution. Additionally, the Bayesian deconvolution method naturally provides a confidence interval, shown by the different samples (red lines) in panel (B) of Figure 2.

For benchmarking purposes and given the relative simplicity of the bimodal normal mixture considered in Figure 2 previously, we next turned a wide variety of synthetic distributions (shown in Figure S1), for which we compared systematically our Bayesian deconvolution method with the FFT-based approach discussed above. To quantity the similarity between the deconvolved and the ground-truth target distribution we used the mean integrated overlap (MIO), as defined in STAR Methods "Efficiency assessment". According to its definition (Equation 108 in STAR Methods), a MIO value of 1 corresponds to a perfect overlap, while the measure is 0 when the two distributions do not overlap at all. We preferred this measure to the more commonly used mean integrated squared error (MISE)^18^^,^^21^^,^^22^^,^^24^ for theoretical reasons, since the MIO measure is easier to interpret, as it corresponds directly to the absolute overlap of two probability distributions. We also avoid other common measures of distribution dissimilarity, such as the Kolmogorov-Smirnov test, since such methods would underestimate the ability of FFT-based methods to converge to the ground-truth deconvolution, given that they lead to artifacts in the resulting distributions, as shown previously.

**Figure 3:**
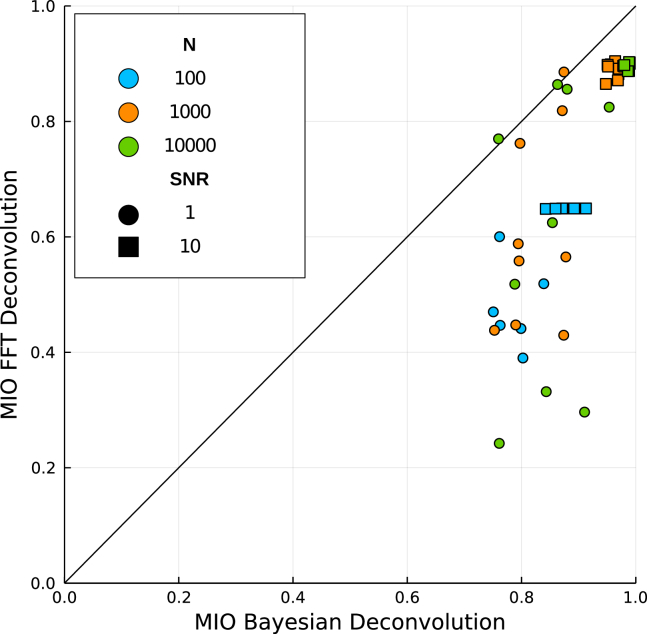
Similarity between the deconvolved and ground-truth target distributions as expressed by the Mean Integrated Overlap (MIO) for the two deconvolution methods (x and y axis), at different sampling sizes for the synthetic datasets shown in Figure S1, with SNR=1 (circles) and SNR=10 (squares) For each value of *N* and SNR, all nine noise and target combinations of Figure S1 are shown.

Figure 3 compares the MIO of the two methods for each of the 9 test cases shown in Figure S1, three different sampling sizes and two noise levels. As can be seen in the figure, the single-cell Bayesian deconvolution method outperforms the Fourier based method in almost all the cases considered, the difference being more substantial for high levels of noise (small SNR, circles). In particular, the overlap is never below 0.7 for the Bayesian methods, while it can reach values as low as 0.2 in the FFT case, depending on the type of distributions involved.

Additionally, it is worth noting that while the single-cell Bayesian deconvolution method is able to reproduce almost perfectly the target distribution (MIO close to 1) for large enough dataset sizes, the Fourier based method always saturates to a suboptimal level even as the number of samples increases, even for low noise levels (large SNR, crosses in Figure 3). Qualitatively, this is due to the oscillatory features in the distribution produced by the noise during the deconvolution with the FFT method, as a consequence of the oscillatory nature of the Fourier basis functions. In general, it has been proved that in FFT methods the convergence to the actual distribution grows sublinearly with the sample size, with scaling dependencies that make the method unfeasible for practical purposes.^18^^,^^21^^,^^27^ Since Bayesian deconvolution makes use of local basis functions, more parsimonious solutions can be obtained, preventing the degradation of the target distribution due to the noise.

In addition to the direct comparison performed between FFT and Bayesian deconvolutions, we compared the results of the deconvolution against a model that simply fits the convolved data ignoring the convolution problem. A condition to impose on any practical deconvolution algorithm is that the result of the deconvolution is not worse than not doing the decomposition at all. The results of this comparison can be observed in Figure S2. The results show that, while the Fourier based method generally tends to make worse predictions than the null model (fitting without deconvolution), the Bayesian approach consistently generates results virtually as good as when the noise is negligible (SNR=10), and improves systematically the distribution in the high-noise case (SNR=1) over the null model. In conclusion, the Bayesian method that we propose has combined benefits, both qualitatively and quantitatively, over other basis methods such as FFT and it consistently improves the result (or at least it does not degrade the quality of the distribution).

### An experimental dataset with external noise

Next, we applied our method to an experimental dataset in which the noise distribution is unknown. To generate a ground truth, we used cells with the Brachyury reporter T/BraGFP in two media conditions (N2B27 supplemented with 3 μM CHIR99 and DMSO as control). At day 3, for each condition, one of the replicas was treated with 20 nM Green CMFDA dye and incubated for 3 min prior to flow cytometry, and the second one was incubated for 3 min with N2B27 (see STAR Methods). Given that the dye incorporates in the cell cytoplasm and its emission spectrum is similar to the one of GFP, the dye acts as a noise source to the GFP signal coming from the Brachyury reporter. Consequently, we have the following four conditions, with their potential outcomes in terms of the signal measured in the FITC-A channel.

(c1) **Control**: Brachyury expression is minimal, and the signal comes mainly from the intrinsic autofluorescence of the cells.
(c2) **Control**+**CMFDA**: Brachyury expression is minimal, and the signal comes mainly from the CMFDA dye, plus intrinsic autofluorescence of the cells.
(c3) **CHIR99**: Brachyury expression is upregulated, and thus the signal contains both the T/BraGFP reporter and the intrinsic autofluorescence of the cells.
(c4) **CHIR99**+**CMFDA**: Brachyury expression is upregulated, and thus the signal contains both the T/BraGFP reporter, the signal from the CMFDA dye and the intrinsic autofluorescence of the cells.

The four signal distributions corresponding to these four conditions are shown in Figure S3. We note that the distribution of the signal coming only from the dye cannot be measured independently, given the intrinsic autofluorescence of the cells. Moreover, the dye might be absorbed differently depending on the state of the cells (CHIR99 treatment), and the low concentration of the dye (∼ nM) might lead to significant cell-to-cell variability on the absorbance of the dye. These features make it a suitable dataset to test the robustness of our method.

**Figure 4:**
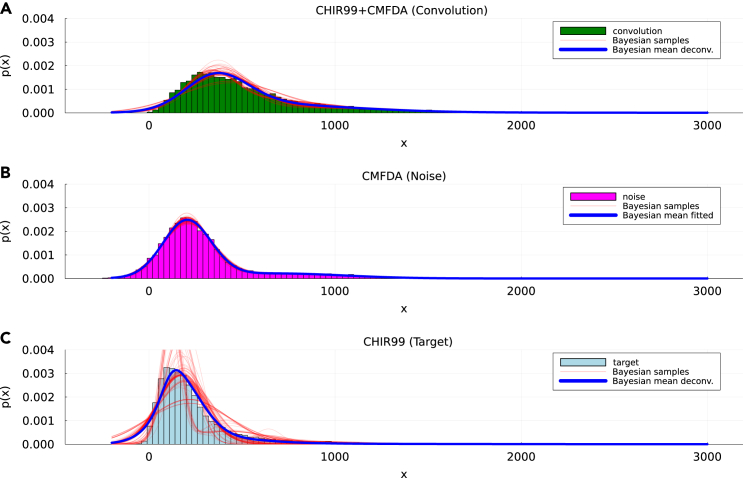
Deconvolution of the CMFDA dye in condition c4 (N2B27+CHIR99+CMFDA) (A–C) Overlaying the distributions we show realizations of the Bayesian sampling process (red lines) for the three distributions (convolution (A), noise (B) and target (C) obtained during the fitting. The convolved and target distributions (green and light blue, respectively) come from the real data. The distribution of the dye (in magenta (B) results from sampling the inferred dye distribution, as described in the text.

We first used our method to deconvolve the signal of the dye from the total signal observed in the experimental conditions (c2), using condition (c1) to define the “noise” distribution. The results of the deconvolution are shown in Figure S4, which presents the dye distribution deconvolved from experimental conditions (c1) and (c2). Once the dye distribution was obtained from the deconvolution of conditions (c1) and (c2), we tested the consistency of our approach by considering the dye signal as the noise in condition (c4). Deconvolving the dye distribution from the one measured in (c4) should lead to the distribution obtained in condition (c3), which we can measure experimentally and thus serves as the ground truth in this case. The result can be seen in Figure 4. The measured signal (top panel) is mildly skewed but distinguishable from a normal distribution (its skewness coefficient is 11.6, while that of a log-normal distribution with the same mean and variance is 5.3). Additionally, we note that here we use a deconvolved dataset for a second deconvolution. Even in these conditions, the inferred distribution resulting from our method is in good agreement with the experimentally measured distribution in condition (c3) (MIO=0.81±0.04) and improves over the null model that ignores the noise (MIO=0.46±0.01). Overall, this experiment shows that our method is robust even when our conditions (target and noise independence) are not fully met, and over other fluctuations present in real datasets.

### Multidimensional distributions

Finally, we applied our method to a multichannel flow cytometry dataset. Specifically, we measured the expression of the mesodermal gene Brachyury through a GFP reporter (T/BraGFP) in mouse embryonic stem cells (see STAR Methods). In this dataset, two populations are expected to appear: a Brachyury-negative population of pluripotent cells, and a positive population capturing the cells exiting from pluripotency toward mesodermal fates.

The Brachyury signal of the GFP reporter was our target signal, and it was acquired through the FITC-A channel. Additionally, we use in what follows five more channels produced by the cytometer, including two light-scattering channels and three extra fluorescence channels (see Figure S5 for scatterplots representing the data in 2-dimensional projections of the 6-dimensional measurement space). These additional channels are barely uncorrelated with the Brachyury signal of interest, and although none of them provide any useful biological signal in our case, we use them to represent potential additional channels to which the deconvolution method described previously can be applied in multidimensional space.

**Figure 5:**
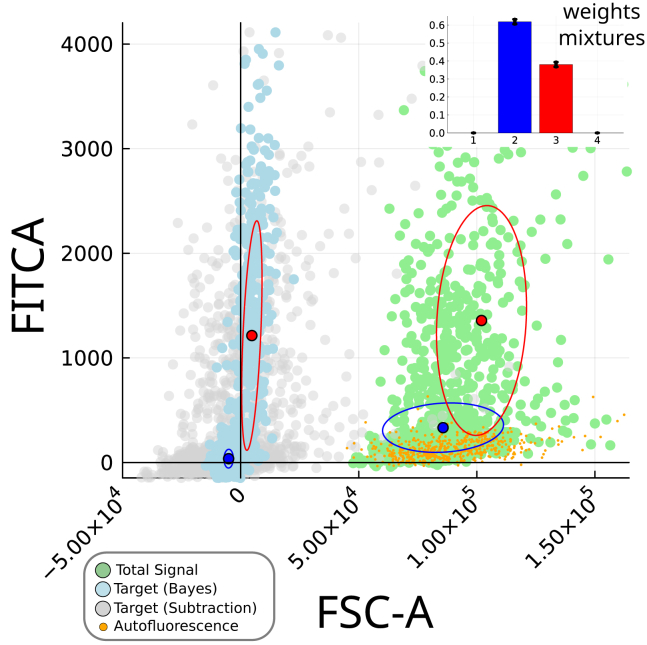
Two-dimensional scatterplot showing a sample of the results of the autofluorescence in a multichannel system Light green circles and orange dots are samples of the real data from the convolved and autofluorescence distributions, respectively. Light blue circles are samples from the results of the deconvolution for the two channels. Light gray circles are the result of subtracting the mean autofluorescence from the convolved distribution, the most traditional approach to correct for autofluorescence. The Gaussian mixture model has two main components in the convolved data (accounting together for more than 95% of the probability density, as seen by the weight contributions of each component shown in the inset), which are shown in red and blue for the convolved and deconvolved distributions.

Figure 5 shows the flow cytometry signal from the T/BraGFP cells (represented as light-green circles) in the plane formed by the Brachyury channel FITC-A and one of the other five channels that were used in the deconvolution. The signal distribution was fitted to a mixture of four normal basis functions, only two of which (2 and 3, see inset in the figure) are enough to reproduce the data. The extent of the two subsets (which we associate with the two populations described previously) is represented by blue and red ellipses that mark one standard deviation away from the means (blue and red solid circles), as determined by the respective covariant matrices in two dimensions (having traced out the other four dimensions).

Brachyury is expressed in the two populations. In particular, there seems to be a non-zero basal expression of the gene in the pluripotent population (the blue ellipse is fully above the horizontal zero axis). These cells, however, are not expected to express Brachyury, as confirmed by the absence of mRNA in the pluripotent states.^33^ Reassuringly, when the data are deconvolved through our Bayesian method, the result shows that the pluripotent population peaks actually around zero (small blue ellipse encompassing the light-blue circles in Figure 5). Similar cases of apparent basal signals in negative cells due to autofluorescence have been reported, such as the false positive detection of MHCII in microglia,^34^ and the false discovery of Foxp3-expressing cell types that are not part of the T cell lineage.^35^

We can compare in this multidimensional case the results of our method with the classical approach of removing the mean value of the autofluorescence from the signal distribution. The autofluorescence data points are shown in orange in Figure 5, while gray circles are the result of removing the mean autofluorescence from the total signal distribution. Whereas this method also corrects the lower peak to zero level expression, the width of the resulting mean distribution is much larger. Other approaches that use additional channels such as regressors^36^ are expected to give results similar to this simpler method, since there are no appropriate regressor channels that can be used to remove the autofluorescence (see STAR Methods "Generalization to multichannel measurements"). Thus, the autofluorescence substraction method and other alternative approaches do not quantify correctly the variance of data. Our method outperforms those other approaches in this regard by using the fact that the variability of FSC-A is a main contribution to the autofluorescence signal, and therefore removing it produces narrower distributions that capture the real variability of the biological phenomenon.

In summary, the Bayesian deconvolution method proposed here is applicable in a straightforward manner to multichannel datasets, and its results are consistent with the expected biological observations. Our method is thus able to correct for artifacts arising from the presence of background signals in multiple detection channels.

## Discussion

In this paper, we propose a Bayesian approach to obtain flow cytometry distributions of protein abundance or activity convolved with a known source of noise. The method, which relies on non-parametric Bayesian techniques, is freely available as a Julia package (https://github.com/dsb-lab/scBayesDeconv.jl) and can be used in a straightforward manner in a purely computational way, without the need of dedicated measurement channels or additional laser sources. It only requires measuring the fluorescently labeled and unlabeled cells and, unlike previously proposed deconvolution methods, it provides well-defined and robust probability distributions, described by mixtures of basis functions.

We measure the quality of the results obtained with our method by comparing the deconvolved distributions with known (ground-truth) target distributions using synthetic data and ad hoc experiments with mouse embryonic stem cells. We argue that the use of local basis distributions to describe all the distributions involved in the problem (both measured and unknown), and the corresponding use of a relatively small number of degrees of freedom, leads to an efficient inference. Finally, the Bayesian nature of the method gives rise to a set of candidate target probability distributions. This reflects the natural indeterminacy present in any process corrupted by noise. The ability to express indeterminacy in the solutions is crucial for any real application of a deconvolution algorithm. We further show that the method is robust and consistent even under strong noise in real datasets.

The approach that we propose here is applicable to flow cytometry datasets with multiple channels, which is the current outcome of modern flow cytometers. Furthermore, the method is consistent with already well-established methods for flow cytometry processing, such as the calculation of spillover matrices and the reduction of autofluorescence using additional channels for autofluorescence regression, as discussed in STAR Methods "Generalization to multichannel measurements". In this sense, our deconvolution approach could be applied to calculate corrected one-fluorophore samples for the calculation of the spillover coefficients, or it could be used after calculating the spillover uncoupling with another method, in order to remove the remaining sources of noise in the dataset.

### Limitations of the study

A potential limitation of the algorithm is the computing time requirements. For $K_{n}$ mixture components of the noise and $K_{t}$ components of the target, a single evaluation of the likelihood function scales as $O\left(K_{n}\times K_{t}\times N\right)$, where *N* is the number of measures in the Bayesian model. The current finite mixture implementation is capable of dealing with datasets of 100000 cells with $K_{n}=4$ and $K_{t}=6$ in about 10 min in a desktop with an i7 processor. This efficiency will be sufficient for most part of the analysis, but can be a drawback for the analysis of very large datasets when compared with non-Bayesian methods. The computational time required by the infinite mixture model depends strongly on the parameter α, which governs the probability of generating new basis functions, but in general this model is more than an order of magnitude slower than its finite counterpart for the same degree of complexity. Handling large datasets is a well-established problem in Bayesian statistics, and some lines of work have explored solutions to reduce this computational cost, which might be worth exploring in the context of the single-cell Bayesian deconvolution method proposed here.^37^^,^^38^^,^^39^

A second potential improvement of the method presented here would be to address the degeneracy under label exchange characteristic of mixture models. Such degeneracy may confound the convergence of the samples to a stationary distribution and the use of the samples for the discovery of clusters, as the same cluster may be assigned to different basis in different samples. A potential solution to this problem would be the implementation of label reassignment methods.^40^

Finally, it would be interesting to generalize this method to other basis functions. While normal distributions have been used in this work because of their well-behaved properties and the existence of analytical results (without losing quality when representing the data), they may not be the most efficient basis functions to describe the skewed distributions typical of flow cytometry datasets. A mixture of gamma or log-normal distributions could adapt to the target datasets using less components than normal distributions. In order to generalize to such basis distributions and keep the efficiency of the method, approximate analytical expressions of the convolved distributions should be derived.

## STAR★Methods

### Key resources table

**Table:** 

REAGENT or RESOURCE	SOURCE	IDENTIFIER
**Chemicals, peptides, and recombinant proteins**		

CHIR99021	Sigma	Cat # SML1046
Activin A	Bio-Techne	Cat # 338-AC
CellTracker Green CMFDA Dye	Thermofisher	Cat # C7025

**Deposited data**		

Flow cytometry data	This paper	https://github.com/dsb-lab/scBayesDeconv.jl
Synthetic data	This paper	https://github.com/dsb-lab/scBayesDeconv.jl

**Experimental models: Cell lines**		

E14 T/Bra::GFP	Gift from Alfonso Martinez-Arias	N/A

**Software and algorithms**		

BD FACSDiva™	BD Biosciences	https://www.bdbiosciences.com/en-us/products/software/instrument-software/bd-facsdiva-software
Python	Python Software Foundation	www.python.org
Julia	Julia Computing	www.julialang.org
scBayesDeconv: A Julia package for noise deconvolution	This paper	https://github.com/dsb-lab/scBayesDeconv.jl

### Resource availability

#### Lead contact

Further information and requests for resources and reagents should be directed to and will be fulfilled by the lead contact, Jordi Garcia-Ojalvo (jordi.g.ojalvo@upf.edu).

#### Materials availability

This study did not generate new unique reagents.

### Experimental model and study participant details

E14 mouse embryonic stem cells containing a knock-in fluorescence reporter for the mesodermal transcription factor Brachyury, T/Bra::GFP were used.^41^ Cells containing the T/Bra::GFP reporter were cultured in ES-Lif (ESL) medium (KnockOut Dulbecco’s Modified Eagle Medium (DMEM) supplemented with 10% fetal bovine serum (FBS), 1x Non-essential aminoacids (NEEA), 50 U/mL Pen/Strep, 1x GlutaMax, 1x Sodium Pyruvate, 50 μM 2-Mercaptoethanol and leukemia inhibitory factor (LIF). Cells adhered to tissue culture-treated 25 cm^2^ (T25) plates (Corning, catalog number 353108) coated with 0.1% gelatin (Millipore, catalog number ES-006-B), and were passaged every second day, as previously described.^42^ Cells were kept at 37°C with 5% ${\text{CO}}_{2}$, and were routinely tested and confirmed to be free from mycoplasma.

Flow cytometry experiments were performed as follows: On day 1, cells were trypsinized and seeded into gelatin-coated 6-well plates (Corning, catalog number 353224) to a final density of $∼10^{5}$ cells/well in 3 mL ESL media. On day 2, the media was replaced by first washing twice with 3 mL ${\text{DPBS}}^{+/+}$ (Phosphate buffered saline containing ${\text{Mg}}^{++}$ and ${\text{Ca}}^{++}$, Sigma, catalog number D8662) and then adding NDiff227 media (N2B27) (Takara Bio, catalog number Y40002)^42^ with the appropriate combination of Brachyury activators: 3 μM CHIR99021 (Sigma, catalog number SML1046) and/or 25 ng/mL Activin A (Bio-Techne, catalog number 338-AC). The same procedure was followed on day 3. For the control conditions, the corresponding volume of dimethyl sulfoxide (DMSO) was added to the medium.

Flow cytometry data was acquired on day 2 or day 3, depending on the type of experiment. For the extrinsic noise experiment, cells were incubated with 1 mL of 20 nM CellTracker Green CMFDA dye (Thermofisher, catalog number C7025) for 3 min prior to trypsinization and flow cytometry. The data was acquired using an LSRIIb flow cytometer, with $2\times 10^{4}$ cells being analyzed per condition. DAPI labelling was used to discard death cells and debris. Furthermore, cell doublets were discarded from the analysis. The readout of protein expression (peak of the signal) was obtained through the FITC-A channel. Measurements were extracted using the BD FACSDiva*™* (BD Biosciences) software and exported in a Python format for subsequent analysis.

### Method details

#### Biological statement of the problem

Flow cytometry provides us with measurements of a target signal *T* in large numbers of single cells. The target signal is emitted by a fluorophore that reports on the abundance (or activity) of a protein of interest within each cell. This signal is affected by autofluorescence (which can be considered a source of noise) produced by elements of the cell other than the fluorophore. Due to the noise, the total signal *C* measured by the device is not directly *T*, but

(Equation 7) C=T+ξ

##### Deconvolving signal from noise

We are interested in the case in which we cannot measure both the signal *C* and the noise ξ independently in the same cell, and thus *T* in that cell cannot be calculated trivially via Equation 7. This limitation is typical of flow cytometry experiments, in which cells can only be measured once. In this case, the only information that can be extracted from the device consists of the distributions of the measured signal, $p_{c}$, and of the background noise by itself, $p_{\xi }$ (by measuring cells without fluorophore), over large populations of cells (tens of thousands in a typical flow cytometry run). We can assume the samples to be independent and identically distributed (iid).

If *T* and ξ and independent of each other, the distributions defined above are related to one another by means of a convolution:

(Equation 8) $$p_{c}\left(C\right)=\int_{0}^{\infty }\int_{0}^{\infty }p_{T}\left(T\right)\;p_{\xi }\left(\xi \right)\;\delta \left(C-\left(T+\xi \right)\right)d\xi \;dT=\int_{0}^{\infty }p_{T}\left(C-\xi \right)\;p_{\xi }\left(\xi \right)d\xi \equiv \left(p_{T}*p_{\xi }\right)\left(C\right)$$

In what follows, we describe a method to extract the distribution $p_{T}$ of the target variable *T* from the observed distributions of *C* and ξ via a deconvolution of Equation 8. The method is applicable to any measurement technique that provides distributions of a signal affected by noise.

##### Generalization to multichannel measurements

Flow cytometry systems have multiple detectors to measure light at different emission frequencies. This allows to target different proteins in the same cell with different fluorophores, measuring their emission at either the peak frequency (classical flow cytometry) or in a set of frequencies that define the emission spectrum (spectral flow cytometry). Independently of the method, the emission of a fluorophore extends over the spectrum, and hence it spills over the different channels. Considering the additive effect of the emission of different fluorophore markers on the measured channels, Equation 7 can be more generally stated as

(Equation 9) $$\begin{array}{cc} c_{k}=\sum_{j=1}^{N_{T}}t_{j}S_{jk}+\xi_{k}\text{,} & k=1\ldots N_{\text{ch}} \end{array}$$

where $c_{k}$, $t_{j}$ and $\xi_{k}$ are realizations of the random variables *C*, *T* and ξ defined in Equation 7 above. The subindex *j* runs over the $N_{T}$ fluorophores (one per target), and the subindex *k* runs over the $N_{\text{ch}}$ channels (so that $\xi_{k}$ represents the contribution of the autofluorescence to channel *k*). The terms $S_{jk}$ define the spillover matrix S, that quantifies how the fluorophore signals are spread over all the measuring channels.

The spillover matrix can be estimated from single fluorophore controls by using regression methods.^36^ To that end, one can perform control experiments in which only one fluorophore is present, and measure the resulting signal in all the channels:

(Equation 10) $$c_{ik}^{\left(j\right)}=t_{ij}^{\left(j\right)}S_{jk}+\xi_{ik}$$

where the subindex *j* now corresponds to the only fluorophore present in the system, the superindex (j) indicates that the experiments were performed in the single-fluorophore condition, and $c_{ik}^{\left(j\right)}$ denotes a total signal in channel *k* coming from cell *i* when only the fluorophore *j* is present in the system. This set of equations is underdetermined, as we do not know neither the real target signal $t_{ij}^{\left(j\right)}$ nor the components $S_{jk}$ of the spillover matrix. However, if we focus for the moment on the channel that corresponds to our single fluorophore (k=j) and impose $S_{jj}=1$, we can write down that $c_{ij}^{\left(j\right)}\approx t_{ij}^{\left(j\right)}$ (ignoring for now the autofluorescence of that channel). This allows us to establish a set of linear regression problems whose solution enables the estimation of the spillover matrix components for which we have single-fluorophore controls:

(Equation 11) $$c_{ik}^{\left(j\right)}\approx c_{ij}^{\left(j\right)}S_{jk}+\xi_{ik}$$

The spillover coefficients $S_{jk}$ can then be estimated using robust regression techniques that remove the noise coming from outliers.^36^

A similar method can be used to reduce the noise coming from the autofluorescence. To that end, one can define an effective “fluorophore signal” $t_{in}$ coming from the autofluorescence, with an associated additional channel *n* not linked to a real fluorophore used in the sample. If the signal in this channel is correlated with the emission of the autofluorescence ξ over the other channels, we can decompose the autofluorescence signal at every channel *k* into a regression term and a noise term:

(Equation 12) $$\begin{array}{ccc} \xi_{ik}\equiv t_{in}S_{nk}+\xi_{ik}^{\prime } & \Rightarrow & c_{ik}=\sum_{j}t_{ij}S_{jk}+t_{in}S_{nk}+\xi_{ik}^{\prime } \end{array}$$

The coefficients $S_{nk}$ indicate how the autofluorescence signal is spread over the other channels. The fluorophore signals and the autofluorescence “signal” can be grouped in a single term:

(Equation 13) $$c_{ik}=\sum_{j=1}^{N_{T}+1}t_{ij}{\bar{S}}_{jk}+\xi_{ik}^{\prime }$$

where now the sum over *j* also includes the autofluorescence signal. We can then calculate the spillover coefficients with the method above, by using the additional channel *n* as an additional ”fluorophore control” for the autofluorescence signal. We also note that writing the autofluorescence as a regression problem in Equation 12 will lead in general to a reduction of the noise in all channels:

(Equation 14) $$\text{Var}\left(\xi_{k}^{\prime }\right)<\text{Var}\left(\xi_{k}\right)$$

Finally, to obtain the original signal, we can multiply Equation 13 by the inverse of the spillover matrix:

(Equation 15) $$C{\bar{S}}^{-1}=T+\xi^{\prime }{\bar{S}}^{-1}\equiv T+\xi^{\prime \prime }$$

We emphasize that this method requires that the autofluorescence signal at the ”noise channel” is sufficiently correlated with its contribution at the other channels, which is not necessarily true *a priori*. Also, Equation 15 shows that the need to deconvolve the noise stands even after the application of the spillover and autofluorescence corrections proposed in the literature. In fact, the deconvolution method that we propose in this article is compatible with existing methods of autofluorescence correction such as the regression method reviewed above, which can be performed before applying our method to the corrected system (Equation 15).

#### Mathematical statement of the problem

Since we do not know the exact underlying distributions, we model them as potentially infinite mixtures of normal basis functions. The Gaussian mixture models for the target and the noise distributions can be represented as

(Equation 16a) $$p\left(t|\omega^{T},\left\{\mu^{T}\right\},\left\{\Sigma^{T}\right\}\right)=\sum_{i=1}^{K_{T}}\omega_{i}^{T}N\left(t|\mu_{i}^{T},\Sigma_{i}^{T}\right)$$

(Equation 16b) $$p\left(\xi |\omega^{\xi },\left\{\mu^{\xi }\right\},\left\{\Sigma^{\xi }\right\}\right)=\sum_{i=1}^{K_{\xi }}\omega_{i}^{\xi }N\left(\xi |\mu_{i}^{\xi },\Sigma_{i}^{\xi }\right)\text{,}$$

where $N\left(x|\mu_{i},\Sigma_{i}\right)$ denotes a normal distribution on the variable vector x (whose components are the different measurement channels), with mean $\mu_{i}$ and variance $\Sigma_{i}$. $K_{T}$ and $K_{\xi }$ represent the number of bases used to describe each distribution, $\omega =\left\{\omega_{i}:i=1,\ldots ,K\right\}$ are their weights, and $\left\{\mu \right\}=\left\{\mu_{i}:i=1,\ldots ,K\right\}$ and $\left\{\Sigma \right\}=\left\{\Sigma_{i}:i=1,\ldots ,K\right\}$ are their characteristic parameters. Note that we have removed the subindices from the distributions of the target and the noise, since according to the mixture representation, $p^{T}$ and $p^{\xi }$ depend exclusively on the parameters defined above.

The mixture decomposition defined in (Equation 16a), (Equation 16b) allows for a flexible and robust representation of unknown and generic distributions. Moreover, exploiting the fact that the convolution of two Gaussian distributions is Gaussian, we have an analytical expression for the distribution of the total variable C:

(Equation 17) $$p\left(c|\varphi^{T},\varphi^{\xi }\right)=\sum_{i=1}^{K_{T}}\sum_{j=1}^{K_{\xi }}\omega_{j}^{T}\omega_{j}^{\xi }N\left(c|\mu_{i}^{T}+\mu_{j}^{\xi },\Sigma_{i}^{T}+\Sigma_{j}^{\xi }\right)$$

where $\varphi^{T}=\left\{\omega^{T},\mu^{T},\Sigma^{T}\right\}$ and $\varphi^{\xi }=\left\{\omega^{\xi },\mu^{\xi },\Sigma^{\xi }\right\}$ represent all the parameters of the target and noise distributions, from which we can parametrize the distribution of the total measured signal. We note that the combination of normal basis function N in Equation 17 above is invariant under changes in the individual basis functions of *T* and ξ, provided the sums of means, $\mu^{T}+\mu^{\xi }$, and variances, $\Sigma^{T}+\Sigma^{\xi }$, are constant.

As discussed in the main text, according to Bayes’ rule, the posterior distribution that represents the probability of the parameters given the data is

(Equation 18) $$p\left(\varphi^{T},\varphi^{\xi }|\left\{c\right\},\left\{\xi \right\}\right)\propto \left[p\left(\left\{c\right\}|\varphi^{T},\varphi^{\xi }\right)p\left(\left\{\xi \right\}|\varphi^{\xi }\right)\right]\left[p\left(\varphi^{\xi }\right)p\left(\varphi^{T}\right)\right]\text{,}$$

where $\left\{c\right\}=\left\{c_{i}:i=1,\ldots ,N_{c}\right\}$ and $\left\{\xi \right\}=\left\{\xi_{i}:i=1,\ldots ,N_{\xi }\right\}$ represent the samples of the total signal and noise, respectively, with the different components of $c_{i}$ and $\xi_{i}$ correspond to the different channels of the cytometer. In Equation 18, the first bracket on the right-hand side corresponds to the likelihood, namely the joint probability of observing the data given the parameters, which we can redefine as

(Equation 19) $$L=p\left(\left\{c\right\}|\varphi^{T},\varphi^{\xi }\right)p\left(\left\{\xi \right\}|\varphi^{\xi }\right)=\prod_{i}^{N_{c}}p\left(c_{i}|\varphi^{T},\varphi^{\xi }\right)\prod_{j}^{N_{\xi }}p\left(\xi_{j}|\varphi^{\xi }\right)\text{,}$$

The form of the likelihood (Equation 19) breaks the symmetry between the target and noise signals, and thus lifts the above-mentioned degeneracy between their parameters exhibited by Equation 17. The second bracket in the right-hand side of Equation 18, in turn, corresponds to the prior distributions of all the parameters of the problem, which we define in what follows.

##### Approximated decomposition of the posterior into two separate problems

It is worth noting that the posterior distribution (Equation 18) can be decomposed as follows:

(Equation 20) $$p\left(\varphi^{T},\varphi^{\xi }|\left\{c\right\},\left\{\xi \right\}\right)=p\left(\varphi^{T}|\varphi^{\xi },\left\{c\right\}\right)p\left(\varphi^{\xi }|\left\{c\right\},\left\{\xi \right\}\right)$$

where we have removed the dependency of the noise signal in the first term of the right-hand side, since only the total signal c defines the parameters of the target distribution, as can be seen from the likelihood (Equation 19). The second term, on the other hand, is conditioned by both the noise and the total signal. Since we usually have as much data for the noise signal {ξ} as for the signal of interest {c}, we can approximately consider that the posterior distribution of the noise mixture parameter $\varphi^{\xi }$ is well represented by the noise data alone:

(Equation 21) $$p\left(\varphi^{T},\varphi^{\xi }|\left\{c\right\},\left\{\xi \right\}\right)\approx p\left(\varphi^{T}|\varphi^{\xi },\left\{c\right\}\right)p\left(\varphi^{\xi }|\left\{\xi \right\}\right)$$

With this approximation, the problem can be decomposed in two separate subproblems: first, finding the probability distribution of the parameters $\varphi^{\xi }$ of the noise mixture; and second, finding the distribution of the parameters $\varphi^{T}$ of the convolution mixture, conditioned on the noise mixture parameters.

In the following section, we go over the mathematical details of the probability distributions that will allow us to sample from the posterior distribution.

#### Relevant probability distributions

In this section, we derive the main expressions that we need to sample our model. Our aim is to have a self-contained derivation of the sampling process of the posterior of a Gaussian normal mixture.

##### Multivariate normal distribution with unknown mean and error

Here we derive the main results of interest on multivariate normal distributions, which we will use when sampling the posterior distribution using normal bases.

###### Likelihood

The multivariate normal distribution for a set of independent identical samples (iid) {x} has the form, up to a scaling parameter,

(Equation 22) $$N\left(\left\{x\right\};\mu ,\Sigma \right)\propto \det\;{\left[\tau \right]}^{n/2}\;\exp\;\left\{-\frac{1}{2}\left[\sum_{i=1}^{n}{\left(x_{i}-\mu \right)}^{T}\tau \left(x_{i}-\mu \right)\right]\right\}$$

where μ is the mean and τ is the precision parameter and *n* is the number of cells being measured. The precision parameter relates to the covariance matrix as

(Equation 23) $$\tau =\Sigma^{-1}$$

It is convenient to rearrange the distribution to make explicit the dependency of the summary statistics:

(Equation 24) $$N\left(\left\{x\right\};\mu ,\Sigma \right)\propto \det\;{\left[\tau \right]}^{n/2}\;\exp\;\left\{-\frac{1}{2}\left[\sum_{i}{\left(x_{i}-\mu \right)}^{T}\tau \left(x_{i}-\mu \right)\right]\right\}\propto \det\;{\left[\tau \right]}^{n/2}\;\exp\;\left\{-\frac{1}{2}\left[\sum_{i}{\left(x_{i}-\bar{x}\right)}^{T}\tau \left(x_{i}-\bar{x}\right)+n{\left(\bar{x}-\mu \right)}^{T}\tau \left(\bar{x}-\mu \right)\right]\right\}\propto \det\;{\left[\tau \right]}^{n/2}\;\exp\;\left\{-\frac{1}{2}\left[n\text{Tr}\left[S_{x}^{2}\tau \right]+n{\left(\bar{x}-\mu \right)}^{T}\tau \left(\bar{x}-\mu \right)\right]\right\}$$

where the summary statistics are the mean,

(Equation 25) $$\bar{x}=\frac{1}{n}\sum_{i}x_{i}$$

and the covariance,

(Equation 26) $$S_{x}^{2}=\frac{1}{n}\sum_{i}\left(x_{i}-\bar{x}\right){\left(x_{i}-\bar{x}\right)}^{T}$$

###### Conjugate prior

A convenient prior for the multivariate distribution is a conjugate prior that allows us to obtain the analytic form of the other distributions:

(Equation 27) $$p\left(\mu |\Sigma \right)=N\left(\mu ;\mu_{0},\kappa_{0}\Sigma \right)$$

(Equation 28) $$p\left(\Sigma \right)=W^{-1}\left(\Sigma ;\Sigma_{0},\nu_{0}\right)$$

where $W^{-1}$ is the inverse Wishart distribution. The hyperparameter $\kappa_{0}$ represents our confidence in the estimation of the mean: the higher $\kappa_{0}$ is, the closer to the mean we will be. The inverse Wishart distribution has the following shape, up to scaling terms:

(Equation 29) $$W^{-1}\left(X;\Sigma ,\nu \right)\propto \frac{\det\;{\left[\Sigma \right]}^{\left(\nu \right)/2}}{\det\;{\left[X\right]}^{\left(\nu +p+1\right)/2}}e^{-\text{Tr}\left[\Sigma X^{-1}\right]/2}$$

where *p* is the number of dimensions. The parameter Σ is the correlation matrix and ν represents our confidence in the estimation of the Σ correlation matrix.

###### Posterior distribution

The posterior distribution will have the following shape:

(Equation 30) $$\begin{array}{l} p\left(\mu ,\Sigma |\left\{x\right\}\right)\propto p\left(\left\{x\right\}|\mu ,\Sigma \right)p\left(\mu |\Sigma ,\kappa_{0},\mu_{0}\right)p\left(\Sigma |\Sigma_{0},\nu_{0}\right)\propto \det\;{\left[\tau \right]}^{n/2}\;\exp\;\left\{-\frac{1}{2}\left[n\text{Tr}\left[S_{x}^{2}\tau \right]+n{\left(\bar{x}-\mu \right)}^{T}\tau \left(\bar{x}-\mu \right)\right]\right\}\times \\ \det\;{\left[\tau \right]}^{1/2}\;\exp\;\left\{-\frac{1}{2}\left[\kappa_{0}{\left(\mu -\mu_{0}\right)}^{T}\tau \left(\mu -\mu_{0}\right)\right]\right\}\det\;{\left[\tau \right]}^{\left(\nu_{0}+p+1\right)/2}\;\exp\;\left\{-\frac{1}{2}\left[\text{Tr}\left[\Sigma_{0}\tau \right]\right]\right\} \end{array}$$

We can use the posterior distribution to obtain different distributions of relevance.

###### Conditional distribution: mean

Retaining the terms involving the mean μ, the conditional distribution of the mean takes the form of a multivariate distribution:

(Equation 31) $$\begin{array}{cc} p\left(\mu |\Sigma ,\left\{x\right\}\right) & \propto \exp\;\left\{-\frac{1}{2}\left[n{\left(\bar{x}-\mu \right)}^{T}\tau \left(\bar{x}-\mu \right)+\kappa_{0}{\left(\mu -\mu_{0}\right)}^{T}\tau \left(\mu -\mu_{0}\right)\right]\right\}\\ & \propto \exp\;\left\{-\frac{1}{2}\left[-2\left(n{\bar{x}}^{T}+\kappa_{0}\mu_{0}\right)\tau \mu +\left(n+\kappa_{0}\right)\mu^{T}\tau \mu \right]\right\}\\ & \propto \exp\;\left\{-\frac{1}{2}\left[{\left(\mu -\tilde{\mu }\right)}^{T}\tilde{\tau }\left(\mu -\tilde{\mu }\right)\right]\right\}\propto N\left(\mu |\tilde{\mu },\tilde{\tau }\right) \end{array}$$

where in the last step we completed the squares. This multivariate normal distribution has effective parameters

(Equation 32) $$\begin{array}{cc} \tilde{\mu }=\frac{n\bar{x}+\kappa_{0}\mu_{0}}{n+\kappa_{0}}\text{,} & \tilde{\tau }=\left(n+\kappa_{0}\right)\tau \end{array}$$

where $\tilde{\mu }$ is a weighted version between the mean statistic and the prior mean, and $\tilde{\tau }$ is an effective precision parameter. The $\kappa_{0}$ factor indicates how close we are from the prior mean.

###### Conditional distribution: covariance

Retaining the terms involving the covariance matrix (and its inverse, the precision matrix), the conditional distribution can be shown to take the form of an inverse Wishart distribution:

p(Σ|μ,{x})∝

$$\det\;{\left[\tau \right]}^{\left(n+\nu_{0}+p+2\right)/2}\;\exp\;\left\{-\frac{1}{2}\left[n\text{Tr}\left[S_{x}^{2}\tau \right]+n{\left(\bar{x}-\mu \right)}^{T}\tau \left(\bar{x}-\mu \right)+\kappa_{0}{\left(\mu -\mu_{0}\right)}^{T}\tau \left(\mu -\mu_{0}\right)+\text{Tr}\left[\Sigma_{0}\tau \right]\right]\right\}$$

$$\propto \det\;{\left[\tau \right]}^{\left(n+\nu_{0}+p+2\right)/2}\;\exp\;\left\{-\frac{1}{2}\left[\text{Tr}\left[\left(nS_{x}^{2}\tau +n\left(\bar{x}-\mu \right){\left(\bar{x}-\mu \right)}^{T}+\kappa_{0}\left(\mu -\mu_{0}\right){\left(\mu -\mu_{0}\right)}^{T}+\Sigma_{0}\right)\tau \right]\right]\right\}$$

(Equation 33) $$\propto \det\;{\left[\tau \right]}^{\left(\tilde{n}+p+1\right)/2}\;\exp\;\left\{-\frac{1}{2}\left[\text{Tr}\left[\tilde{\Sigma }\tau \right]\right]\right\}\propto W^{-1}\left(\Sigma |\tilde{\Sigma },\tilde{n}\right)$$

where the effective parameters are

(Equation 34) $$\tilde{n}=n+\nu_{0}+1$$

(Equation 35) $$\tilde{\Sigma }=nS_{x}^{2}+n\left(\bar{x}-\mu \right){\left(\bar{x}-\mu \right)}^{T}+\kappa_{0}\left(\mu -\mu_{0}\right){\left(\mu -\mu_{0}\right)}^{T}+\Sigma_{0}$$

In this last expression, it is worth noting that each term represents a different kind of uncertainty: the first term is the uncertainty coming from the mean statistic, the second corresponds to the uncertainty with which the mean statistic represents the actual mean, the third term is the uncertainty coming from the prior mean, and the last term is the uncertainty coming from the prior itself.

###### Marginal distribution: covariance

One additional distribution that we will need is the marginal distribution of the variance:

(Equation 36) $$p\left(\Sigma |\left\{x\right\}\right)=\int p\left(\Sigma ,\mu |{\left\{x_{i}\right\}}_{i}\right)d\mu \propto \int \det\;{\left[\tau \right]}^{n/2}\;\exp\;\left\{-\frac{1}{2}\left[n\text{Tr}\left[S_{x}^{2}\tau \right]+n{\left(\bar{x}-\mu \right)}^{T}\tau \left(\bar{x}-\mu \right)\right]\right\}\times \det\;{\left[\tau \right]}^{1/2}\;\exp\;\left\{-\frac{1}{2}\left[\kappa_{0}{\left(\mu -\mu_{0}\right)}^{T}\tau \left(\mu -\mu_{0}\right)\right]\right\}\det\;{\left[\tau \right]}^{\left(\nu_{0}+p+1\right)/2}\;\exp\;\left\{-\frac{1}{2}\left[\text{Tr}\left[\Sigma_{0}\tau \right]\right]\right\}d\mu \propto \det\;{\left[\tau \right]}^{\left(n+\nu_{0}+p+2\right)/2}\;\exp\;\left\{-\frac{1}{2}\left[n\text{Tr}\left[S_{x}^{2}\tau \right]+\text{Tr}\left[\Sigma_{0}\tau \right]\right]\right\}\times \left[\int \exp\;\left\{-\frac{1}{2}\left[\underset{\left(*\right)}{\underset{︸}{\kappa_{0}{\left(\mu -\mu_{0}\right)}^{T}\tau \left(\mu -\mu_{0}\right)+n{\left(\bar{x}-\mu \right)}^{T}\tau \left(\bar{x}-\mu \right)}}d\mu \right]\right\}\right]$$

Reorganizing the elements in (∗),

(Equation 37) $$\left(*\right)=\left(\kappa_{0}+n\right)\mu^{T}\tau \mu -2\mu^{T}\tau \left(\kappa_{0}\mu_{0}+n\bar{x}\right)+\left(n{\bar{x}}^{T}\tau \bar{x}+\kappa_{0}\mu_{0}\tau \mu_{0}\right)$$

(Equation 38) $$={\left(\mu -\tilde{\mu }\right)}^{T}\tilde{\tau }\left(\mu -\tilde{\mu }\right)\underset{\left(**\right)}{\underset{︸}{-{\tilde{\mu }}^{T}\tilde{\tau }\tilde{\mu }+\left(n{\bar{x}}^{T}\tau \bar{x}+\kappa_{0}\mu_{0}\tau \mu_{0}\right),}}$$

where we have defined,

(Equation 39) $$\tilde{\mu }=\frac{\kappa_{0}\mu_{0}+n\bar{x}}{\kappa_{0}+n}$$

(Equation 40) $$\tilde{\tau }=\left(\kappa_{0}+n\right)\tau \text{,}$$

we can further regroup the part in (∗∗).

(Equation 41) $$\begin{array}{c} \left(**\right)=\frac{-\kappa_{0}^{2}\mu_{0}^{T}\tau \mu_{0}-n^{2}{\bar{x}}^{T}\tau \bar{x}-2\kappa_{0}n\mu_{0}^{T}\tau \bar{x}+n^{2}{\bar{x}}^{T}\tau \bar{x}+\kappa_{0}^{2}\mu_{0}\tau \mu_{0}+n\kappa_{0}{\bar{x}}^{T}\tau \bar{x}+\kappa_{0}n\mu_{0}\tau \mu_{0}}{\kappa_{0}+n}\\ \frac{\kappa_{0}n\left({\bar{x}}^{T}\tau \bar{x}-2\mu_{0}^{T}\tau \bar{x}+\mu_{0}\tau \mu_{0}\right)}{\kappa_{0}+n}=\frac{\kappa_{0}n}{\kappa_{0}+n}{\left(\bar{x}-\mu_{0}\right)}^{T}\tau \left(\bar{x}-\mu_{0}\right) \end{array}$$

Inserting these terms

(Equation 42) $$\begin{array}{cc} p\left(\Sigma |\left\{x\right\}\right) & \propto \det\;{\left[\tau \right]}^{\left(n+\nu_{0}+p+2\right)/2}\;\exp\;\left\{-\frac{1}{2}\left[\text{Tr}\left[\left(nS_{x}^{2}+\Sigma_{0}+\frac{\kappa_{0}n}{\kappa_{0}+n}\left(\bar{x}-\mu_{0}\right){\left(\bar{x}-\mu_{0}\right)}^{T}\right)\tau \right]\right]\right\}\\ & \times \left[\int \exp\;\left\{-\frac{1}{2}\left[{\left(\mu -\tilde{\mu }\right)}^{T}\tilde{\tau }\left(\mu -\tilde{\mu }\right)\right]\right\}d\mu \right] \end{array}$$

now we can calculate the integral as a multivariate normal:

(Equation 43) $$p\left(\Sigma |\left\{x\right\}\right)\propto \propto \det\;{\left[\tau \right]}^{\left(n+\nu_{0}+p+2\right)/2}\;\exp\;\left\{-\frac{1}{2}\left[\text{Tr}\left[\left(nS_{x}^{2}+\Sigma_{0}+\frac{\kappa_{0}n}{\kappa_{0}+n}\left(\bar{x}-\mu_{0}\right){\left(\bar{x}-\mu_{0}\right)}^{T}\right)\tau \right]\right]\right\}\det\;{\left[\tilde{\tau }\right]}^{-1/2}\propto \det\;{\left[\tau \right]}^{\left(n+\nu_{0}+p+1\right)/2}\;\exp\;\left\{-\frac{1}{2}\left[\text{Tr}\left[\tilde{\Sigma }\tau \right]\right]\right\}\propto W^{-1}\left(\Sigma |\tilde{\Sigma },\tilde{n}\right)$$

where in the last step we use the fact that $\det\tilde{\tau }\propto \det\;\tau$ and the effective parameters of the inverse Wishart distribution are

(Equation 44) $$\tilde{n}=n+\nu_{0}+1$$

(Equation 45) $$\tilde{\Sigma }=nS_{x}^{2}+\frac{\kappa_{0}n}{\kappa_{0}+n}\left(\bar{x}-\mu_{0}\right){\left(\bar{x}-\mu_{0}\right)}^{T}+\Sigma_{0}$$

###### Posterior predictive distribution

One last distribution of interest is the probability of a new data point given the already observed set of observations:

(Equation 46) $$\begin{array}{cc} p\left(y|\left\{x\right\}\right) & =\iint p\left(y|\mu ,\Sigma \right)p\left(\mu ,\Sigma |{\left\{x_{i}\right\}}_{i}\right)d\mu d\Sigma \propto \iint \det\;{\left[\tau \right]}^{\left(n+\nu_{0}+p+3\right)/2}\\ & \times \exp\;\left\{-\frac{1}{2}\left[\underset{\left(*\right)}{\underset{︸}{{\left(y-\mu \right)}^{T}\tau \left(y-\mu \right)+n{\left(\bar{x}-\mu \right)}^{T}\tau \left(\bar{x}-\mu \right)+\kappa_{0}{\left(\mu -\mu_{0}\right)}^{T}\tau \left(\mu -\mu_{0}\right)}}\right]\right\}\\ & \times \exp\;\left\{-\frac{1}{2}\left[\text{Tr}\left[\left(nS_{x}^{2}+\Sigma_{0}\right)\tau \right]\right]\right\}d\mu d\Sigma \end{array}$$

We can rearrange the elements in (∗), completing squares as we did when calculating the mean conditional distribution (Multivariate normal distribution with unknown mean and error):

(Equation 47) $$\left(*\right)=\underset{\left(**\right)}{\underset{︸}{n{\bar{x}}^{T}\tau \bar{x}+y^{T}\tau y+\kappa_{0}\mu_{0}^{T}\tau \mu_{0}-\tilde{\mu }\tilde{\tau }{\tilde{\mu }}^{\mu }}}+{\left(\mu -{\tilde{\mu }}^{\mu }\right)}^{T}\tilde{\tau }\left(\mu -{\tilde{\mu }}^{\mu }\right)\text{,}$$

where we have retained all the terms involving τ and μ this time, as they are necessary for integrating out. The effective parameters are now

(Equation 48) $${\tilde{\mu }}^{\mu }=\frac{n\bar{x}+\kappa_{0}\mu_{0}+y}{n+\kappa_{0}+1}$$

(Equation 49) $${\tilde{\tau }}^{\mu }=\left(n+\kappa_{0}+1\right)\tau$$

We can further rearrange the terms in (∗∗) to obtain an expression with the *y* in quadrature:

$$\left(**\right)=n{\bar{x}}^{T}\tau \bar{x}+y^{T}\tau y+\kappa_{0}\mu_{0}^{T}\tau \mu_{0}-\frac{1}{n+\kappa_{0}+1}{\left(n\bar{x}+\kappa_{0}\mu_{0}+y\right)}^{T}\tau \left(n\bar{x}+\kappa_{0}\mu_{0}+y\right)$$

$$=\frac{1}{n+\kappa_{0}+1}\left[\left(n{\bar{x}}^{T}\tau \bar{x}+y^{T}\tau y+\kappa_{0}\mu_{0}^{T}\tau \mu_{0}\right)\left(n+\kappa_{0}+1\right)-{\left(n\bar{x}+\kappa_{0}\mu_{0}+y\right)}^{T}\tau \left(n\bar{x}+\kappa_{0}\mu_{0}+y\right)\right]$$

$$=\frac{1}{n+\kappa_{0}+1}\left[n{\left(y-\bar{x}\right)}^{T}\tau \left(y-\bar{x}\right)+\kappa_{0}{\left(y-\mu_{0}\right)}^{T}\tau \left(y-\mu_{0}\right)+n\kappa_{0}{\left(\mu_{0}-\bar{x}\right)}^{T}\tau \left(\mu_{0}-\bar{x}\right)\right]$$

$$=\frac{\left(n+\kappa_{0}\right)}{n+\kappa_{0}+1}{\left(y-{\tilde{\mu }}^{y}\right)}^{T}\tau \left(y-{\tilde{\mu }}^{y}\right)+\frac{\left(\frac{n\kappa_{0}}{n+\kappa_{0}}+n\kappa_{0}\right)}{n+\kappa_{0}+1}{\left(\mu_{0}-\bar{x}\right)}^{T}\tau \left(\mu_{0}-\bar{x}\right)$$

(Equation 50) $$=m^{0}{\left(y-{\tilde{\mu }}^{y}\right)}^{T}\tau \left(y-{\tilde{\mu }}^{y}\right)+m^{1}{\left(\mu_{0}-\bar{x}\right)}^{T}\tau \left(\mu_{0}-\bar{x}\right)$$

where in the second to the third step we group by quadrature and complete squares to group the terms with *y*, and then group by quadrature the terms on the left. The effective parameters are

(Equation 51) $$\begin{array}{ccc} {\tilde{\mu }}^{y}=\frac{\left(n\bar{x}+\kappa_{0}\mu_{0}\right)}{n+\kappa_{0}}\text{,} & m^{0}=\frac{n+\kappa_{0}}{n+\kappa_{0}+1}\text{,} & m^{1}=\frac{\frac{n\kappa_{0}}{n+\kappa_{0}}+n\kappa_{0}}{n+\kappa_{0}+1} \end{array}$$

We can now insert the obtained term in the original expression:

(Equation 52) $$\begin{array}{l} p\left(y|\left\{x\right\}\right)\propto \iint \det\;{\left[\tau \right]}^{\left(n+\nu_{0}+p+3\right)/2}\times \\ \exp\;\left\{-\frac{1}{2}\left[m^{0}{\left(y-{\tilde{\mu }}^{y}\right)}^{T}\tau \left(y-{\tilde{\mu }}^{y}\right)+m^{1}{\left(\mu_{0}-\bar{x}\right)}^{T}\tau \left(\mu_{0}-\bar{x}\right)+\text{Tr}\left[nS_{x}^{2}\tau \right]+\text{Tr}\left[\Sigma_{0}\tau \right]\right]\right\}\times \\ \exp\;\left\{-\frac{1}{2}\left[{\left(\mu -{\tilde{\mu }}^{\mu }\right)}^{T}{\tilde{\tau }}^{\mu }\left(\mu -{\tilde{\mu }}^{\mu }\right)\right]\right\}d\mu d\Sigma =\int \det\;{\left[\tau \right]}^{\left(n+\nu_{0}+p+3\right)/2}\;\exp\;\left\{-\frac{1}{2}\left[\text{Tr}\left[\tilde{\Sigma }\tau \right]\right]\right\}\times \\ \left[\int \exp\;\left\{-\frac{1}{2}\left[{\left(\mu -{\tilde{\mu }}^{\mu }\right)}^{T}{\tilde{\tau }}^{\mu }\left(\mu -{\tilde{\mu }}^{\mu }\right)\right]\right\}d\mu \right]d\Sigma \end{array}$$

where the effective covariance has the form,

(Equation 53) $$\tilde{\Sigma }=m^{0}\left(y-{\tilde{\mu }}^{y}\right){\left(y-{\tilde{\mu }}^{y}\right)}^{T}+m^{1}\left(\mu_{0}-\bar{x}\right){\left(\mu_{0}-\bar{x}\right)}^{T}+nS_{x}^{2}+\Sigma_{0}$$

The mean parameter in Equation 52 only appears in the term in brackets, so we can integrate it in a straightforward manner as a multivariate normal integral:

(Equation 54) $$\begin{array}{l} \begin{array}{l} p\left(y|\left\{x\right\}\right)\propto \int \det\;{\left[\tau \right]}^{\left(n+\nu_{0}+p+3\right)/2}\;\exp\;\left\{-\frac{1}{2}\left[\text{Tr}\left[\tilde{\Sigma }\tau \right]\right]\right\}\det\;{\left[\tilde{\tau }\right]}^{-1/2}d\Sigma \end{array}\\ \begin{array}{l} \propto \int \det\;{\left[\tau \right]}^{\left(n+\nu_{0}+p+2\right)/2}\;\exp\;\left\{-\frac{1}{2}\left[\text{Tr}\left[\tilde{\Sigma }\tau \right]\right]\right\}\;d\Sigma \end{array} \end{array}$$

where $\det\left[{\tilde{\tau }}^{\mu }\right]\propto \det\left[\tau \right]$. The last integral is an inverse Wishart that we can integrate directly, leading to

(Equation 55) $$p\left(y|\left\{x\right\}\right)\propto \det\;{\left[\tilde{\Sigma }\right]}^{-\left(n+\nu_{0}+1\right)/2}$$

We can now reorganize the effective covariance. If we define the matrix

(Equation 56) $${\tilde{\Sigma }}^{y}=\frac{1}{m^{0}}\left(m^{1}\left(\mu_{0}-\bar{x}\right){\left(\mu_{0}-\bar{x}\right)}^{T}+nS_{x}^{2}+\Sigma_{0}\right)\text{,}$$

we can rewrite the effective covariance $\tilde{\Sigma }$ as

(Equation 57) $$\tilde{\Sigma }=\left(\left(y-{\tilde{\mu }}^{y}\right){\left(y-{\tilde{\mu }}^{y}\right)}^{T}{\tilde{\tau }}^{y}+I\right)m_{0}{\tilde{\Sigma }}^{y}\text{,}$$

Insert this expression in Equation 55 we obtain

(Equation 58) $$\begin{array}{c} p\left(y|\left\{x\right\}\right)\propto \det\left[\left(\left(y-{\tilde{\mu }}^{y}\right){\left(y-{\tilde{\mu }}^{y}\right)}^{T}{\tilde{\tau }}^{y}+I\right)n_{0}{\tilde{\Sigma }}^{y}\right]\\ \propto \det\;{\left[\left(y-{\tilde{\mu }}^{y}\right){\left(y-{\tilde{\mu }}^{y}\right)}^{T}{\tilde{\tau }}^{y}+I\right]}^{-\left(n+\nu_{0}+1\right)/2}={\left|1+{\left(y-{\tilde{\mu }}^{y}\right)}^{T}{\tilde{\tau }}^{y}\left(y-{\tilde{\mu }}^{y}\right)\right|}^{-\left(n+\nu_{0}+1\right)/2}\\ ={\left|1+\frac{1}{\tilde{\nu }}{\left(y-{\tilde{\mu }}^{y}\right)}^{T}\left(\tilde{\nu }{\tilde{\tau }}^{y}\right)\left(y-{\tilde{\mu }}^{y}\right)\right|}^{-\left(\tilde{\nu }+p\right)/2}=t_{\tilde{\nu }}\left(y;{\tilde{\mu }}^{y},{\tilde{\Sigma }}^{y}/\tilde{\nu }\right) \end{array}$$

which is a multivariate T-distribution with

(Equation 59) $$\begin{array}{ccc} m^{0}=\frac{n+\kappa_{0}}{n+\kappa_{0}+1}\text{,} & m^{1}=\frac{\frac{n\kappa_{0}}{n+\kappa_{0}}+n\kappa_{0}}{n+\kappa_{0}+1}\text{,} & \tilde{\nu }=n+\nu_{0}+1-p \end{array}$$

(Equation 60) $${\tilde{\mu }}^{y}=\frac{\left(n\bar{x}+\kappa_{0}\mu_{0}\right)}{n+\kappa_{0}}$$

(Equation 61) $${\tilde{\Sigma }}^{y}=\frac{1}{m^{0}}\left(m^{1}\left(\mu_{0}-\bar{x}\right){\left(\mu_{0}-\bar{x}\right)}^{T}+nS_{x}^{2}+\Sigma_{0}\right)$$

##### Finite mixture distributions

We derive in this section the statistics relevant to mixture models.

###### Likelihood

The likelihood of a finite mixture model with *K* components has the form

(Equation 62) $$p\left(\left\{x\right\}|\left\{\varphi \right\},\omega \right)=\prod_{i}\left(\sum_{j}^{K}\omega_{j}p\left(x_{i}|\varphi_{j}\right)\right)\text{,}$$

where the vector sets $\left\{x\right\}=\left\{x_{i}:i=1,\ldots ,N\right\}$ and $\left\{\varphi \right\}=\left\{\varphi_{j}:j=1\ldots K\right\}$ run over the number of samples (cells) *N* and the number of mixture components *K*, respectively (in what follows we use the subindices *i* and *j* with those two distinct meanings) (We remind the reader that the dimension of the vectors $x_{i}$ and $\varphi_{j}$ is equal to the number of measurement channels.). The set of parameters ω are called the weights of the mixture model, and $p\left(\left\{x\right\}|\varphi_{j}\right)$ are the base distributions. We can extend this model to introduce a set of hidden indicator variables {z}, defined as

(Equation 63) $$z_{ij}=\delta_{jk_{i}}$$

for some $k_{i}\in \left\{1,\ldots ,K\right\}$, and where $\delta_{ij}$ is the Kronecker delta. This variable basically tells from which distribution $p\left(\left\{x\right\}|\varphi_{i}\right)$ the variable came from. Using this set of hidden variables, our model (Equation 62) can be rewritten as

(Equation 64) $$p\left(\left\{x\right\},\left\{z\right\}|\left\{\varphi \right\},\omega \right)=\prod_{i}^{N}\prod_{j}^{K}p{\left(x_{i}|\varphi_{j}\right)}^{z_{ij}}\omega_{j}^{z_{ij}}$$

It is straightforward to see that, if we take the marginal distribution over the hidden variables, we recover the original distribution:

(Equation 65) $$p\left(\left\{x\right\}|\left\{\varphi \right\},\omega \right)=\prod_{i}^{N}\left(\sum_{j}^{K}p\left(x_{i},z_{ij}=1|\varphi_{j},\omega \right)\right)=\prod_{i}^{N}\left(\sum_{j}^{K}p\left(x_{i}|\varphi_{j}\right)\omega_{j}\right)$$

where only when the indicator variable is one of the corresponding term survives. The use of indicator variables makes it possible to compute analytically the posterior distribution of the mixture model, as the base distributions are now in product form. The hidden indicator variables are not known, thus we will have to sample from them as well.

###### Conjugate prior

A conjugate prior for the mixture model is

(Equation 66) p(ω|α)=Dirichlet(ω|α)

where the Dirichlet distribution has the form

(Equation 67) $$\text{Dirichlet}\left(\omega |\alpha \right)\propto \prod_{i}^{K}\omega_{i}^{\alpha_{i}-1}$$

The hyperprior parameters α are usually set to be symmetrical and to scale with the number of mixture components:

(Equation 68) $$\begin{array}{cc} \alpha_{i}=\alpha /K & \forall i \end{array}$$

In this way, the prior distribution only depends on one hyperparameter α that indicates the strength from the uniform weights.

###### Posterior distribution

Putting together the likelihood and the prior distribution, the posterior of the mixture model is

(Equation 69) $$p\left(\left\{\varphi \right\},\omega |\left\{x\right\},\left\{z\right\}\right)\propto \prod_{j}^{K}\left(\prod_{i}p{\left(x_{i}|\varphi_{j}\right)}^{z_{ij}}\omega_{j}^{z_{ij}}\right)\omega_{j}^{\alpha /K-1}p\left(\varphi_{j}^{0}\right)\text{,}$$

where the last term is the set of priors for each base distribution.

###### Conditional distribution: weights

Taking the terms from the posterior that involve the weights:

(Equation 70) $$p\left(\omega |\left\{z\right\}\right)\propto \prod_{j}^{K}\left(\prod_{i}\omega_{j}^{z_{ij}}\right)\omega_{j}^{\alpha /K-1}=\text{Dirichlet}\left(\omega |\tilde{n}\right)$$

where ${\tilde{n}}_{j}=\sum_{i}z_{ij}+\alpha$.

###### Conditional distribution: indicator variables

As we already mentioned, the indicator variables are not known, so we have to sample from them too. As we are considering identically independent samples, we can obtain the conditional distribution from each indicator variable independently as

(Equation 71) $$p\left(c_{i}|\omega ,\left\{\varphi \right\},x_{i}\right)\propto \prod_{j}^{K}p{\left(x_{i}|\varphi_{j}\right)}^{z_{ij}}\omega_{j}^{z_{ij}}=\text{Multinomial}\left(z_{i};1,{\left\{p\left(x_{i}|\varphi_{j}\right)\omega_{j}\right\}}_{j}\right)$$

It is worth noting that the indicator will be sampled from a particular base distribution for the weights of that base, but also by how well that sample lies inside the base distribution.

###### Conditional distribution: base distribution parameters

Finally, because the base distributions are in product form due to the introduction of the indicator variables, the parameters of each base can be computed independently as

(Equation 72) $$p\left(\varphi_{j}|\left\{x\right\},\left\{z\right\}\right)\propto p\left({\left\{x\right\}}_{j}|\varphi_{j}\right)p\left(\varphi_{j}^{0}\right)$$

where ${\left\{x\right\}}_{j}$ represents the subsample of cells whose indicator variable belongs to the corresponding mixture component.

##### Infinite mixture distributions

In this section we define basic results from infinite Dirichlet processes (for an insightful tutorial see Li et al.^32^) that allow us to consider infinite mixtures.

###### Taking the infinite limit

In order to take the limit to infinite clusters, we need to remove the dependence from the number of clusters in a mixture model. For that, we need to remove the dependence on the weights of our probability distribution. Consider for the moment a basis distribution that is uniform in space. The joint probability distribution conditioned on the priors would be

(Equation 73) p({z},ω|α)=p({z}|ω)p(ω|α)

where the first term in the right-hand side is the likelihood of the indicator variables as in Equation 64, also given in Equation 71, which is a multinomial distribution. The second term is the prior distribution of the mixture distribution (Equation 67), which is a Dirichlet distribution. From this expression, we can calculate the marginal distribution of the indicator variables conditioned to the prior parameter:

(Equation 74) $$\begin{array}{cc} p\left(\left\{z\right\}|\alpha \right) & =\int p\left(\left\{z\right\}|\omega \right)p\left(\omega |\alpha \right)d\omega =\int \prod_{i}\left(\prod_{j}\omega_{j}^{z_{ij}}\right)\frac{\Gamma \left(\alpha \right)}{\Gamma {\left(\alpha /K\right)}^{K}}\prod_{j}\omega_{j}^{\alpha /K-1}d\omega \\ & =\frac{\Gamma \left(\alpha \right)}{\Gamma {\left(\alpha /K\right)}^{K}}\int \prod_{j}\omega_{j}^{n_{j}+\alpha /K-1}d\omega =\frac{\Gamma \left(\alpha \right)}{\Gamma {\left(\alpha /K\right)}^{K}}\frac{\prod_{j}\Gamma \left(n_{j}+\alpha /K\right)}{\Gamma \left(n+\alpha /K\right)} \end{array}$$

where the third equality makes use of the statistic $n_{j}=\sum_{i}z_{ij}$. The integral can be identified as a multinomial distribution without the scaling factor.

The expression above still contains explicitly the dependence on the number of components in the mixture *K*, and thus its limit K→∞ cannot be computed in a straightforward manner. To have a more amenable expression, let us consider that we take out the sample *l* and reassign it to a new cluster. The probability of this sample to be assigned to any of the clusters, conditioned on all the other indicator variables, is

(Equation 75) $$\begin{array}{cc} p\left(z_{lk}=1|{\left\{z\right\}}_{\neg l},\alpha \right) & =\frac{p\left({\left\{z\right\}}_{\neg l},z_{lk}=1|\alpha \right)}{p(p\left({\left\{z\right\}}_{\neg l}|\alpha \right)}=\frac{\frac{\Gamma \left(\alpha \right)}{\Gamma {\left(\alpha /K\right)}^{K}}\frac{\prod_{j}\Gamma \left(n_{j|l}+\delta_{jk}+\alpha /K\right)}{\Gamma \left(n+\alpha /K\right)}}{\frac{\Gamma \left(\alpha \right)}{\Gamma {\left(\alpha /K\right)}^{K}}\frac{\prod_{j}\Gamma \left(n_{j|l}+\alpha /K\right)}{\Gamma \left(n+\alpha /K-1\right)}}\\ & =\frac{\Gamma \left(n+\alpha /K-1\right)}{\Gamma \left(n+\alpha /K\right)}\prod_{j}\frac{\Gamma \left(n_{j|l}+\delta_{jk}+\alpha /K\right)}{\Gamma \left(n_{j|l}+\alpha /K\right)}=\frac{n_{k|l}+\alpha /K}{n+\alpha -1}\text{,} \end{array}$$

where have made explicit the range of cells over which the samples {z} are taken in each case. To that end, we define ${\left\{z\right\}}_{\neg l}\equiv {\left\{z\right\}}_{i\in \left(1,\ldots ,l-1,l+1,\ldots n\right)}$ and $n_{\neg l}\equiv \sum_{i\in \left(1,\ldots ,l-1,l+1,\ldots n\right)}z_{ij}$ to refer to the subsets that contain all elements except *l*. With this expression, it is straightforward to take the limit to infinite components:

(Equation 76) $$p_{\infty }\left(z_{lk}=1|{\left\{z\right\}}_{\neg l},\alpha \right)=\lim_{K\to \infty }p\left(z_{lk}=1|{\left\{z\right\}}_{\neg l},\alpha \right)=\frac{n_{\neg l}}{n+\alpha -1}$$

We now have the probability that a sample is assigned to a base that has other indicator variables. Let us now consider for the moment that we have *K* bases with assigned samples. The probability that a sample is assigned to a new base will be:

(Equation 77) $$\begin{array}{cc} p_{\infty }\left(z_{l,K+1}=1|{\left\{z\right\}}_{\neg l},\alpha \right) & =1-\sum_{j=1}^{K}p_{\infty }\left(z_{lk}=1|{\left\{z\right\}}_{\neg l},\alpha \right)\\ & =1-\sum_{j=1}^{K}\frac{n_{k|l}}{n+\alpha -1}=1-\frac{n-1}{n+\alpha -1}=\frac{\alpha }{n+\alpha -1} \end{array}$$

There is a non-zero probability that the cell will be assigned to a new base that was not populated before, and the probability of populating this new cluster will depend on the hyperparameter α.

###### Adding a non-uniform basis

The results above have been derived considering a uniform basis distribution. In the most general case, the basis will be non-uniform. From Equation 64 it is very easy to see that if we had a non-uniform distribution, the corresponding term will drop out of the integral (Equation 74). Proceeding in the same way as before, all the terms in Equation 75 will cancel out, except $p\left(x_{l}|\varphi_{k}\right)$. The probabilities of a new assignation will be

(Equation 78) $$p_{\infty }\left(z_{lk}=1|{\left\{z\right\}}_{\neg l},\left\{\varphi \right\},\alpha \right)\propto \frac{n_{\neg l}}{n+\alpha -1}p\left(x_{i}|\varphi_{k}\right)$$

for a basis with a populated sample and

(Equation 79) $$p_{\infty }\left(z_{l,K+1}=1|{\left\{z\right\}}_{\neg l},\left\{\varphi \right\},\alpha \right)\propto \frac{\alpha }{n+\alpha -1}p\left(x_{i}|\varphi^{0}\right)$$

for the creation of a new basis.

###### Using the predictive posterior distribution

Similar way to the approach used in Multivariate normal distribution with unknown mean and error, we can directly predict the new outcomes in the case of an infinite mixture as a function of already observed data. The probabilities of a new assignation will be

(Equation 80) $$p_{\infty }\left(z_{lk}=1|{\left\{z\right\}}_{\neg l},\left\{\varphi \right\},\alpha \right)\propto \frac{n_{\neg l}}{n+\alpha -1}p\left(x_{i}|{\left\{x\right\}}_{k}\right)$$

for a basis with a populated sample and

(Equation 81) $$p_{\infty }\left(z_{l,K+1}=1|{\left\{z\right\}}_{\neg l},\left\{\varphi \right\},\alpha \right)\propto \frac{\alpha }{n+\alpha -1}p\left(x_{i}|\varphi^{0}\right)$$

for a new basis.

##### Modifications of the convolution distribution

Introducing indicator variables as indicated in Finite mixture distributions, the convolution of two multivariate mixture distributions like the one described by Equation 17 takes the form

(Equation 82) $$p\left(\left\{x\right\},\left\{z\right\}|\left\{\varphi^{\xi }\right\},\omega^{\xi },\left\{\varphi^{T}\right\},\omega^{T}\right)=\prod_{i}^{N}\prod_{j}^{K^{\xi }}\prod_{k}^{K^{T}}N{\left(x_{i}|\mu_{j}^{\xi }+\mu_{k}^{T},\Sigma_{j}^{\xi }+\Sigma_{k}^{T}\right)}^{z_{ijk}}{\left(\omega_{j}^{\xi }\omega_{k}^{T}\right)}^{z_{ijk}}$$

where the indicator variable is now 1 if the sample *i* belongs to the noise base *j* and target base *k*. Considering the approximation described in Approximated decomposition of the posterior into two separate problems, the only sampling parameters that we have to go over will be $\left\{\varphi^{T}\right\}$, the weights $\omega^{T}$ and the indicator variables z. The main challenge is that there is no close form to group all the terms involving $\Sigma^{T}$ in single effective distributions as the ones derived in Multivariate normal distribution with unknown mean and error. In order to get a tractable expression, we would like to transform the convolved covariances in such a way that the following expression follows:

(Equation 83) $$M{\left(\Sigma_{j}^{\xi }+\Sigma_{k}^{T}\right)}^{-1}=\left(M+A\right)\Sigma_{k}^{T-1}$$

for given matrices A, M. Isolating, A we obtain that

(Equation 84) $$A=-M{\left(\Sigma_{j}^{\xi }+\Sigma_{k}^{T}\right)}^{-1}\Sigma_{j}^{\xi }$$

Now, in most practical cases we can consider that the convolved covariance will be close to the expected covariance matrix, and we can approximate the expression above by

(Equation 85) $$A∼-M{\hat{\tau }}_{jk}\Sigma_{j}^{\xi }\text{,}$$

where the effective expected covariance is

(Equation 86) $${\hat{\Sigma }}_{jk}=n_{jk}S_{jk}^{2}+\kappa_{0}\left({\bar{x}}_{jk}-\mu_{0}\right){\left({\bar{x}}_{jk}-\mu_{0}\right)}^{T}+\Sigma_{0}$$

All the results derived in the preceding sections take into account this approximation:

(Equation 87) $$\mu \to \mu^{T}+\mu^{\xi }$$

(Equation 88) $$\Sigma \to \Sigma^{T}+\Sigma^{\xi }$$

(Equation 89) $$\tau^{T}\to \left(I-{\hat{\tau }}_{jk}\Sigma_{j}^{\xi }\right)\tau^{T}$$

(Equation 90) $$z_{ij}\to z_{ijk}$$

The weights of the target distribution (Finite mixture distributions) will be computed using the sum over the samples for all the noise samples

(Equation 91) $$n_{k}^{T}=\sum ijz_{ijk}$$

Sampling from the mean and covariance parameters in the distribution needs, however, a closer look.

###### Prior distribution

In order for the prior to be conjugated, we have to scale the prior distribution in terms of the prior of the noise distribution.

(Equation 92) $$p\left(\mu |\tilde{\Sigma }\right)=N\left(\mu ;\mu_{0},\kappa_{0}\tilde{\Sigma }\right)$$

(Equation 93) $$p\left(\tilde{\Sigma }\right)=W^{-1}\left(\tilde{\Sigma };\Sigma_{0},\Sigma^{\xi },\nu_{0}\right)$$

where the effective covariance is

(Equation 94) $$\tilde{\Sigma }=\Sigma^{T}+\Sigma^{\xi }$$

###### Conditional distribution: covariance

If we focus the analysis in a single set of target parameters $\varphi_{k}$, the posterior probability of the variance and the mean has the form

(Equation 95) $$p\left(\varphi_{k}^{T}|\left\{\varphi^{\xi }\right\},\left\{x\right\},\left\{z\right\}\right)\propto \prod_{i}^{N}\prod_{j}^{K_{\xi }}N{\left(x_{i}|\mu_{j}^{\xi }+\mu_{k}^{T},\Sigma_{j}^{\xi }+\Sigma_{k}^{T}\right)}^{z_{ijk}}p\left(\mu |\Sigma_{j}^{\xi }+\Sigma_{k}^{T}\right)p\left(\Sigma_{j}^{\xi }+\Sigma_{k}^{T}\right)$$

In contrast with the case without convolution, the normal distributions cannot be grouped together in a simple distribution that can be sampled by standard procedures. We can group the data coming from a specific dataset,

(Equation 96) $$p\left(\varphi_{k}^{T}|\left\{\varphi^{\xi }\right\},\left\{x\right\},\left\{z\right\}\right)\propto \det\;{\left(\Sigma_{j}^{\xi }+\Sigma_{k}^{T}\right)}^{-\left(n_{jk}+\nu_{0}+1\right)/2}\;\exp\;\left\{-\frac{1}{2}\left[{\tilde{\Sigma }}_{jk}{\left(\Sigma_{j}^{\xi }+\Sigma_{k}^{T}\right)}^{-1}\right]\right\}$$

where the variance is

(Equation 97) $${\tilde{\Sigma }}_{jk}=n_{jk}S_{jk}^{2}+n_{jk}\left({\bar{x}}_{jk}-\mu_{j}^{\xi }-\mu_{k}^{T}\right){\left(\bar{x}-\mu_{j}^{\xi }-\mu_{k}^{T}\right)}^{T}+\kappa_{0}\left(\mu_{k}^{T}-\mu_{0}\right){\left(\mu_{k}^{T}-\mu_{0}\right)}^{T}+\Sigma_{0}$$

Within the proposed approximation, we can now group all the different distributions:

(Equation 98) $${\tilde{\Sigma }}_{k}=\sum_{j}{\tilde{\Sigma }}_{jk}\left(I-{\hat{\tau }}_{jk}\Sigma_{j}^{\xi }\right)$$

and we can sample new covariance matrices for the target with the inverse Wishart distribution.

(Equation 99) $$\Sigma_{k}^{T}∼W^{-1}\left({\tilde{\Sigma }}_{k},n_{k}+\nu_{0}+1\right)$$

###### Conditional distribution: mean

The mean distribution can be easily computed, regrouping all the terms in the following summary statistics:

$$\begin{array}{l} {\tilde{\tau }}_{k}=\sum_{j}\left(n_{jk}+\kappa_{0}\right){\left(\Sigma_{j}^{\xi }+\Sigma_{k}^{T}\right)}^{-1}\\ {\tilde{\mu }}_{k}={\tilde{\Sigma }}_{j}\left(\sum_{j}{\left(\Sigma_{j}^{\xi }+\Sigma_{k}^{T}\right)}^{-1}\left(n_{jk}{\bar{x}}_{jk}+\kappa_{0}\mu_{0}\right)\right)\text{,} \end{array}$$

and sampling from a multivariate normal,

(Equation 100) $$\mu_{k}^{T}∼N\left({\tilde{\mu }}_{k},{\tilde{\tau }}_{k}\right)$$

#### Hyperparameter selection

Our approach the has five hyperparameters:

• α: Determines how close we are from a uniform distribution of weights (finite mixtures), or the potential of generating a new basis (infinite mixture).
• $\mu_{0}$: The center of the prior normal distribution $\mu_{0}$.
• $\kappa_{0}$: The confidence we have of being close to $\mu_{0}$.
• $\Sigma_{0}$: The covariance of the prior normal distribution.
• $\nu_{0}$: The confidence we have of being close to $\sigma_{0}$.

Flow cytometry applications have in general large datasets, making the approach quite insensitive to the choice of α, $\kappa_{0}$ and $\nu_{0}$, as the statistics will dominate the model. The choice of mean and covariance matrix, however, depend on the data distribution. Appropriate estimators of these parameters are the mean and the covariance matrix of the whole dataset. This imposes a soft-informative prior over the region where the density should lie. In cases where the distribution has fat tails, the density prior can be very flat because of the outliers, making it difficult for the algorithm to find the correct distribution as the prior spreads over the basis components. In these cases, narrower prior covariance matrices can help to fit the model correctly.

#### Gibbs sampling algorithms

In this section, we describe the algorithms to sample the noise distribution according to the approximation described in Approximated decomposition of the posterior into two separate problems.

##### Efficient updating of summary statistics

Once we have computed the mean or the variance over a set of samples {x} and we add or remove a single sample, it is possible to update the statistic without having to recompute the metric fully over the new set, which in general will be more time-consuming as we will have to go over a sum over all the size of the set. If we remove a sample:

(Equation 101) $$n_{j}^{rem}=n_{j}-1$$

(Equation 102) $${\bar{x}}_{j}^{rem}=\frac{n_{j}{\bar{x}}_{j}-x_{i}}{n_{j}^{rem}}$$

(Equation 103) $$S_{j}^{2rem}=\frac{1}{n_{j}^{rem}}\left(n_{j}S_{j}^{2}+n_{j}{\bar{x}}_{j}{\bar{x}}_{j}^{T}-x_{i}x_{i}^{T}\right)-{\bar{x}}_{j}^{rem}{\bar{x}}_{j,rem}^{T}$$

On the other hand, if we add a sample:

(Equation 104) $$n_{j}^{add}=n_{j}+1$$

(Equation 105) $${\bar{x}}_{j}^{add}=\frac{n_{j}{\bar{x}}_{j}+x_{i}}{n_{j}^{add}}$$

(Equation 106) $$S_{j}^{2add}=\frac{1}{n_{j}^{add}}\left(n_{j}S_{j}^{2}+n_{j}{\bar{x}}_{j}{\bar{x}}_{j}^{T}+x_{i}x_{i}^{T}\right)-{\bar{x}}_{j}^{new}{\bar{x}}_{j,add}^{T}$$

##### Finite normal mixture distribution

The procedure in this case is as follows:

0 Initialize the parameters.
  0.1 Initialize the indicator variables {z}: Assign to each cell one of the *K* normal distributions using any initialization procedure (random assignment, k-means…).
  0.2 Compute the statistics of each base:

$$\begin{array}{ccc} n_{j}=\sum_{i}z_{ij} & {\bar{x}}_{j}=\frac{1}{n_{j}}\sum_{i}z_{ij}x_{i} & S_{j}^{2}=\frac{1}{n_{j}}\sum_{i}z_{ij}x_{i}x_{i}^{T}-{\bar{x}}_{j}{\bar{x}}_{j}^{T} \end{array}$$

0.3 Initialize parameters of each base:

$$\begin{array}{ccc} \mu_{j}={\bar{x}}_{j} & \Sigma_{j}=S_{j}^{2} & \omega_{j}=\frac{1}{n}\sum_{i}z_{ij} \end{array}$$

where $n=\sum_{j}n_{j}$ is the size of the dataset.

1 Sampling. For *N* iterations do
  1.1 Sample indicator variables as in Finite mixture distributions. For each sample *i*:
    1.1.1 Compute weights: $w_{j}=\omega_{j}p\left(x_{i}|\varphi_{j}\right)$.
    1.1.2 Sample new indicator: z∼Multinomial(1,w).
  1.2 Sample weights as from Finite mixture distributions. For each base j∈1,…K do
    1.2.1 Compute $\tilde{n}$: ${\tilde{n}}_{j}=\sum_{i}z_{ij}+\alpha /K$
    1.2.2 Sample new weights: $\omega ∼\text{Dirichlet}\left(\tilde{n}\right)$
  1.3 Sample variances and mean. For each base j∈1,…K do
    1.3.1 Compute summary statistics:

$$\begin{array}{ccc} n_{j}=\sum_{i}z_{ij} & {\bar{x}}_{j}=\frac{1}{n_{j}}\sum_{i}z_{ij}x_{i} & S_{j}^{2}=\frac{1}{n_{j}}\sum_{i}z_{ij}x_{i}x_{i}^{T}-{\bar{x}}_{j}{\bar{x}}_{j}^{T} \end{array}$$

1.3.2 Sample new covariance (Multivariate normal distribution with unknown mean and error):

$$\begin{array}{l} {\tilde{n}}_{j}=n_{j}+\nu_{0}+1\\ {\tilde{\Sigma }}_{j}=n_{j}S_{j}^{2}+n_{j}\left(\bar{x}-\mu_{j}\right){\left(\bar{x}-\mu_{j}\right)}^{T}+\kappa_{0}\left(\mu_{j}-\mu_{0}\right){\left(\mu_{j}-\mu_{0}\right)}^{T}+\Sigma_{0}\\ \Sigma_{j}∼W^{-1}\left({\tilde{n}}_{j},{\tilde{\Sigma }}_{j}\right) \end{array}$$

1.3.2 Sample new mean (Multivariate normal distribution with unknown mean and error):

$$\tilde{\mu }=\frac{n_{j}{\bar{x}}_{j}+\kappa_{0}\mu_{0}}{n_{j}+\kappa_{0}}$$

$$\tilde{\tau }=\left(n_{j}+\kappa_{0}\right)\tau_{j}$$

$$\mu_{j}∼N\left(\tilde{\mu },\tilde{\Sigma }\right)$$

##### Infinite normal mixture distribution

The approach in this case has the following steps:

0 Initialize the parameters.
  0.1 Initialize the indicator variables {z}: Assign to each cell one of the *K* normal distributions using any initialization procedure (random assignment, k-means…).
  0.2 Compute the statistics of each base:

$$\begin{array}{ccc} n_{j}=\sum_{i}z_{ij} & {\bar{x}}_{j}=\frac{1}{n_{j}}\sum_{i}z_{ij}x_{i} & S_{j}^{2}=\frac{1}{n_{j}}\sum_{i}z_{ij}x_{i}x_{i}^{T}-{\bar{x}}_{j}{\bar{x}}_{j}^{T} \end{array}$$

0.3 Initialize parameters of each base:

$$\begin{array}{ccc} \mu_{j}={\bar{x}}_{j} & \Sigma_{j}=S_{j}^{2} & \omega_{j}=\frac{1}{n}\sum_{i}z_{ij} \end{array}$$

where $n=\sum_{j}n_{j}$ is the size of the dataset.

1 Sampling. For *N* iterations, do
  1.1 Reassign samples. For each sample i∈{1,…,n}
    1.1.1 Remove sample *i.* Consider sample $z_{ij}=1$.

If $n_{j}=1$ (the only sample assigned to that base distribution), remove the distribution from the active basis.

Otherwise recompute the summary statistics for base distribution *j* removing one sample as described in Efficient updating of summary statistics.

1.1.2 Compute weights (not-normalized) for reassigning the sample as described in Multivariate normal distribution with unknown mean and error and Infinite mixture distributions.

For the active bases j∈{1,…K}.

$$\begin{array}{ll} m_{j}^{0}=\frac{n_{j}+\kappa_{0}}{n_{j}+\kappa_{0}+1} & m_{j}^{1}=\frac{\frac{n_{j}\kappa_{0}}{n_{j}+\kappa_{0}}+n_{j}\kappa_{0}}{n_{j}+\kappa_{0}+1}\\ {\tilde{\mu }}_{j}^{y}=\frac{\left(n_{j}\bar{x}+\kappa_{0}\mu_{0}\right)}{n_{j}+\kappa_{0}} & {\tilde{\Sigma }}_{j}^{y}=\frac{1}{m^{0}}\left(m^{1}\left(\mu_{0}-\bar{x}\right){\left(\mu_{0}-\bar{x}\right)}^{T}+n_{j}S_{x}^{2}+\Sigma_{0}\right)\\ w_{j}=\frac{n_{j}}{n+\alpha -1}t_{\tilde{\nu }}\left(y;{\tilde{\mu }}^{y},{\tilde{\Sigma }}^{y}/\tilde{\nu }\right) & \end{array}$$

and for creating a new basis,

$$w_{K+1}=\frac{\alpha }{n+\alpha -1}N\left(x_{i}|\mu_{0}^{y},\Sigma_{0}^{y}\right)$$

1.1.3 Sample new indicator: $z_{i}^{new}∼\text{Multinomial}\left(1,w\right)$.
1.1.4 Update statistics.

If $z_{iK+1}=1$ create a new basis distribution and assign new statistics to it,

$$\begin{array}{ccc} n_{K+1}=1 & {\bar{x}}_{K+1}=x_{i} & S_{K+1}^{2}=0 \end{array}$$

else, update the statistics adding a term as described in Efficient updating of summary statistics.

1.2 Sample basis parameters. For each basis j∈{1,…,K}:

1.2.1 Sample the weights (Finite mixture distributions):

$$\begin{array}{cc} {\tilde{n}}_{j}=n_{j}+\alpha & \omega ∼\text{Dirichle}t\left(\tilde{n}\right) \end{array}$$

1.2.2 Sample the covariance matrix (Multivariate normal distribution with unknown mean and error):

$$\begin{array}{cc} {\tilde{n}}_{j}=n_{j}+\nu_{0}+1 & {\tilde{\Sigma }}_{j}=nS_{j}^{2}+\frac{\kappa_{0}n_{j}}{\kappa_{0}+n_{j}}\left({\bar{x}}_{j}-\mu_{0}\right){\left({\bar{x}}_{j}-\mu_{0}\right)}^{T}+\Sigma_{0} \end{array}$$

$$\Sigma_{j}∼W^{-1}\left({\tilde{\Sigma }}_{j},{\tilde{n}}_{j}\right)$$

1.2.3 Sample the mean (Multivariate normal distribution with unknown mean and error):

$$\begin{array}{cc} {\tilde{\mu }}_{j}=\frac{n_{j}{\bar{x}}_{j}+\kappa_{0}\mu_{0}}{n_{j}+\kappa_{0}} & {\tilde{\Sigma }}_{j}=\Sigma_{j}/\left(n_{j}+\kappa_{0}\right) \end{array}$$

$$\mu_{j}∼N\left({\tilde{\mu }}_{j},{\tilde{\Sigma }}_{j}\right)$$

##### Modifications to the convolved distribution

The preceding algorithms are the sampling algorithms for non-convolved finite and infinite mixture sampling. The convolved cases are exactly the same, but changing the equations with the modifications described in Modifications of the convolution distribution. In addition to this, it is necessary to add a step in the sampling loop to sample new parameters from the already fitted noise distribution ${\left\{\varphi_{j}^{\xi }\right\}}_{j}$.

#### Efficiency assessment

So far, the efficiency of deconvolution methods has been assessed using quantifiers applicable to point estimates, which are the ones proposed in the literature to date. To compare the efficiency of our model with previous methods that do not obey the positivity nor the normalization conditions, we can use the Mean Integrated Squared Estimation (MISE) measure:

(Equation 107) $$\text{MISE}=\int_{-\infty }^{\infty }{\left(p_{\text{true}}\left(x\right)-p_{\text{est}}\left(x\right)\right)}^{2}dx\text{,}$$

where $p_{\text{true}}$ is the real target distribution and $p_{\text{est}}$ is the point estimate of the deconvolved distribution. This is the traditional measure of convergence, but it is hard to interpret as it only has a lower bound. To address this issue, we introduce the mean integrated overlap (MIO):

(Equation 108) $$\text{MIO}=1-\frac{1}{2}\int_{-\infty }^{\infty }\left|p_{\text{true}}\left(x\right)-p_{\text{est}}\left(x\right)\right|dx$$

This measure has the property that it is bounded in the interval [0,1] if the true and the estimated distributions are normalized, with 0 corresponding to the case of no overlap between distributions, and 1 to the case of complete overlap. Values below zero can be obtained if the estimated distribution does not follow the positivity requirement nor the normalization condition, as it is the case in FFT-based deconvolutions.

#### Synthetic data

For the target distribution we generated samples from three distributions: symmetric bimodal, asymmetric bimodal and skew symmetric distributions (Figure S1). This choice of distributions intends to capture the features present in real datasets, such as the presence of multiple peaks, different cluster sizes, and the generally non-Gaussian character of the data.^27^ As for the noise, we generated a set of three different noise datasets containing normal, skewed, and Student’s t distributions (the last two of which exhibit fat tails) (Figure S1), in order to test the flexibility of the method against very dissimilar autofluorescence profiles. In order to check the impact of the noise strength, the convolutions between target and noise were generated at two signal-to-noise ratios (SNR). In our context, we define the SNR as the ratio between target and signal variances for the whole dataset. We chose a case with negligible noise (SNR=10), and a difficult case where the noise is of the same magnitude as the signal (SNR=1). We generated sample datasets of different sizes, with 100, 1000 and, 10000 samples. The high range of these values is representative of typical single-cell flow cytometry experiments. The combination of the different target distribution types, noise distribution types, SNRs, and sample sizes generates a collection of 54 datasets with known ground truth.

#### Software

The software pipeline presented here, including MCMC for finite and infinite normal mixtures, has been implemented as a Julia package (scBayesDeconv.jl). The source code, with manual compilation and installation instructions, as well as full documentation and a notebook with examples of use, can be obtained publicly from GitHub (https://github.com/dsb-lab/scBayesDeconv.jl).

## Data Availability

• Experimental data have been deposited at https://github.com/dsb-lab/scBayesDeconv.jl and are publicly available as of the date of submission. • All original code has been deposited at https://github.com/dsb-lab/scBayesDeconv.jl and is publicly available as of the date of submission. • Any additional information required to reanalyze the data reported in this paper is available from the lead contact upon request.
