# Supplementary material for: Single-cell Bayesian deconvolution
Source: iScience. 2023 Sep 19;26(10):107941. doi: 10.1016/j.isci.2023.107941 (PMC10579429; doi:10.1016/j.isci.2023.107941)
Supplement: Document S1. Figures S1–S5 [file mmc1.pdf]

iScience, Volume *26*

Supplemental information

Single-cell Bayesian deconvolution

Gabriel Torregrosa-Cortés, David Oriola, Vikas Trivedi, and Jordi Garcia-Ojalvo

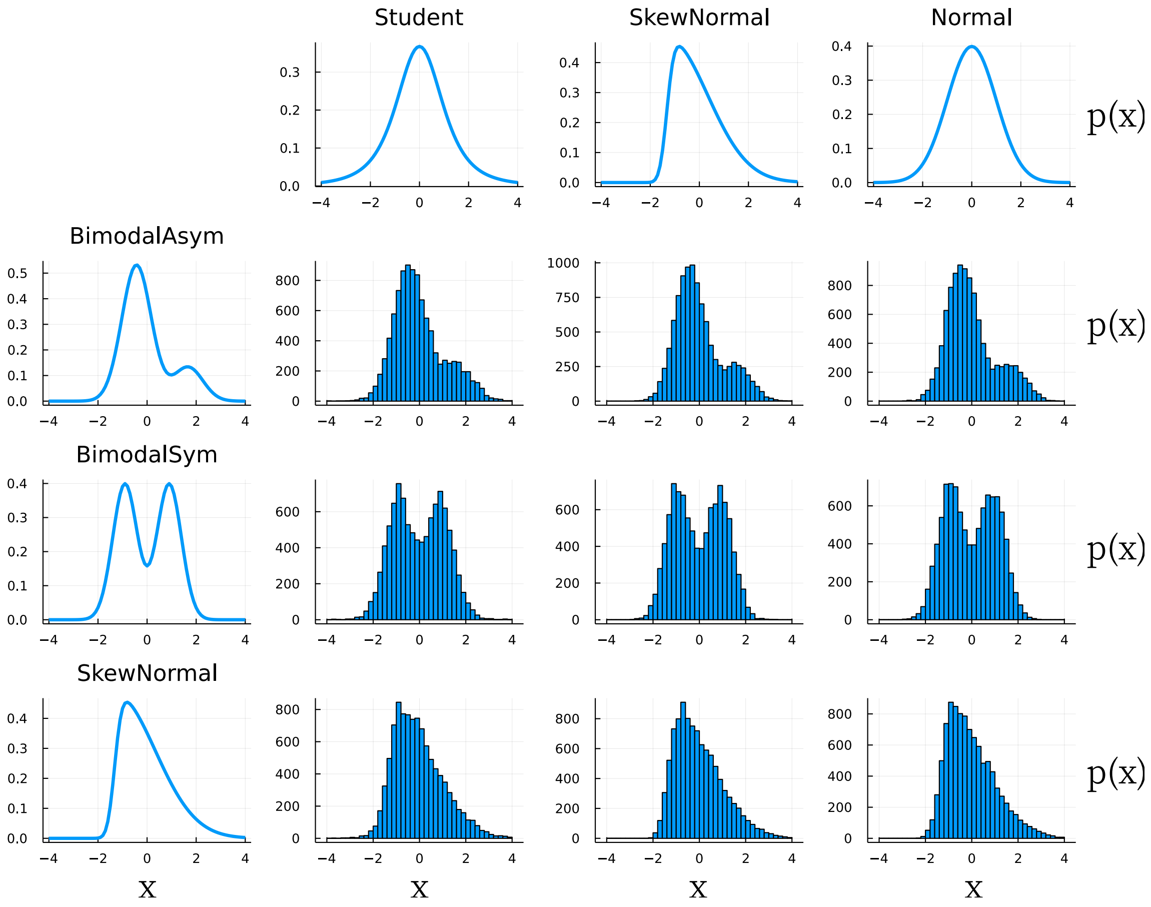

Figure S1: **Synthetic data, Related to Figures 2 and 3.** Synthetic target distributions (left) and noise distributions (top) and the resulting convolutions for a SNR=2.

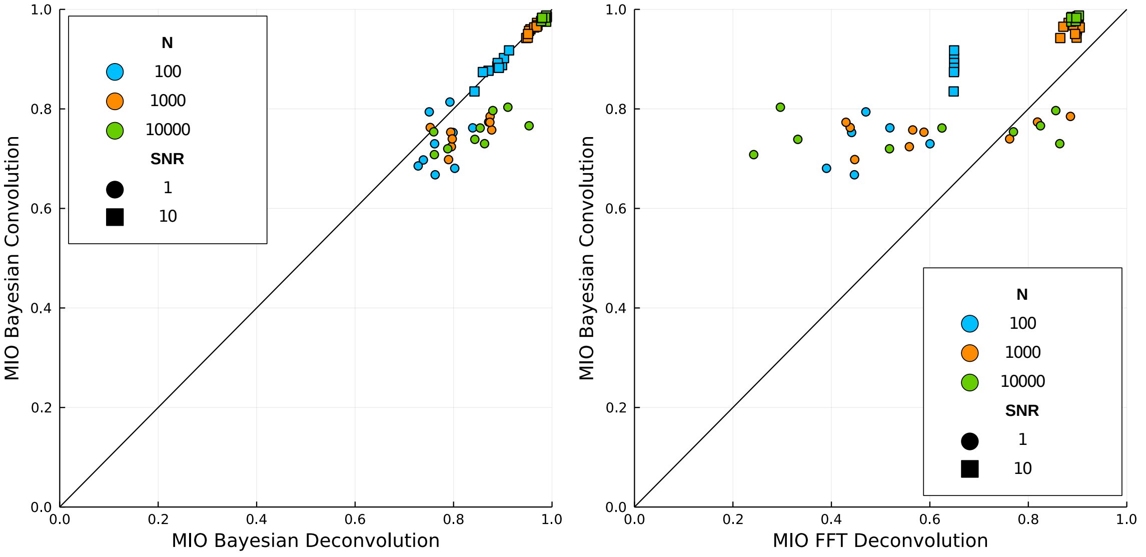

Figure S2: **Comparing the Bayesian and FFT methods, Related to Figure 3.** Comparison between the deconvolved and ground-truth target distributions as expressed by the Mean Integrated Overlap (MIO) for the Bayesian (left) and FFT (right) methods (x axis), with the results of a null Bayesian model fitting directly to the convolved data (y axis), ignoring the noise.

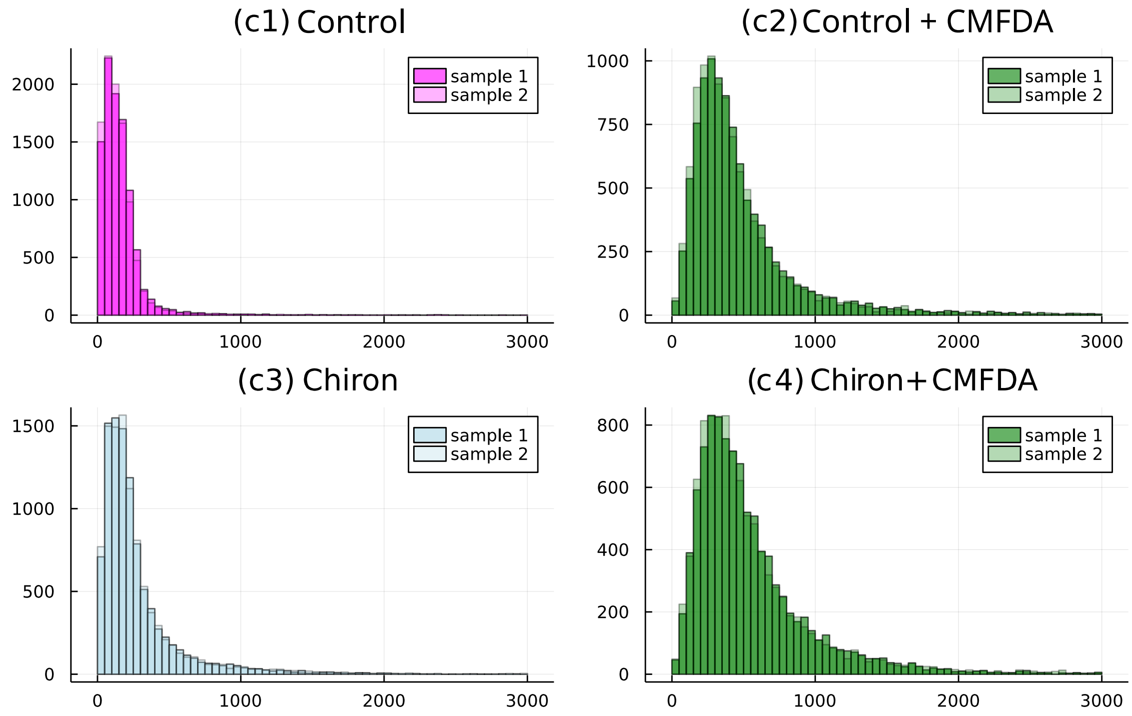

Figure S3: **Experimental data, Related to Figure 4.** Flow cytometry distributions obtained in the four experimental conditions discussed in Sec. III.B of the main text.

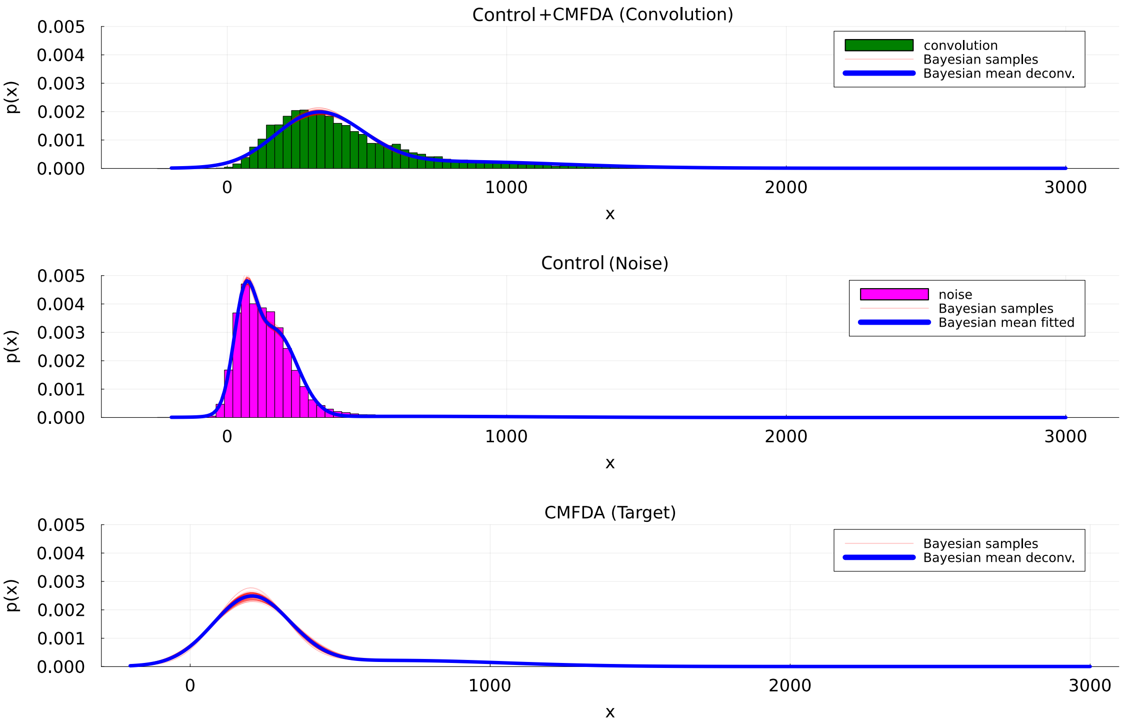

Figure S4: **Intermediate deconvolutions of the experimental data, Related to Figure 4.** Flow cytometry distributions corresponding to the data conditions c2 (top panel in green) and c1 (middle panel in magenta) discussed in Sec. III.B. Overlayed on the distributions we show realizations of the Bayesian sampling process (red lines) for the three distributions (noise, convolution and target) obtained during the fitting.

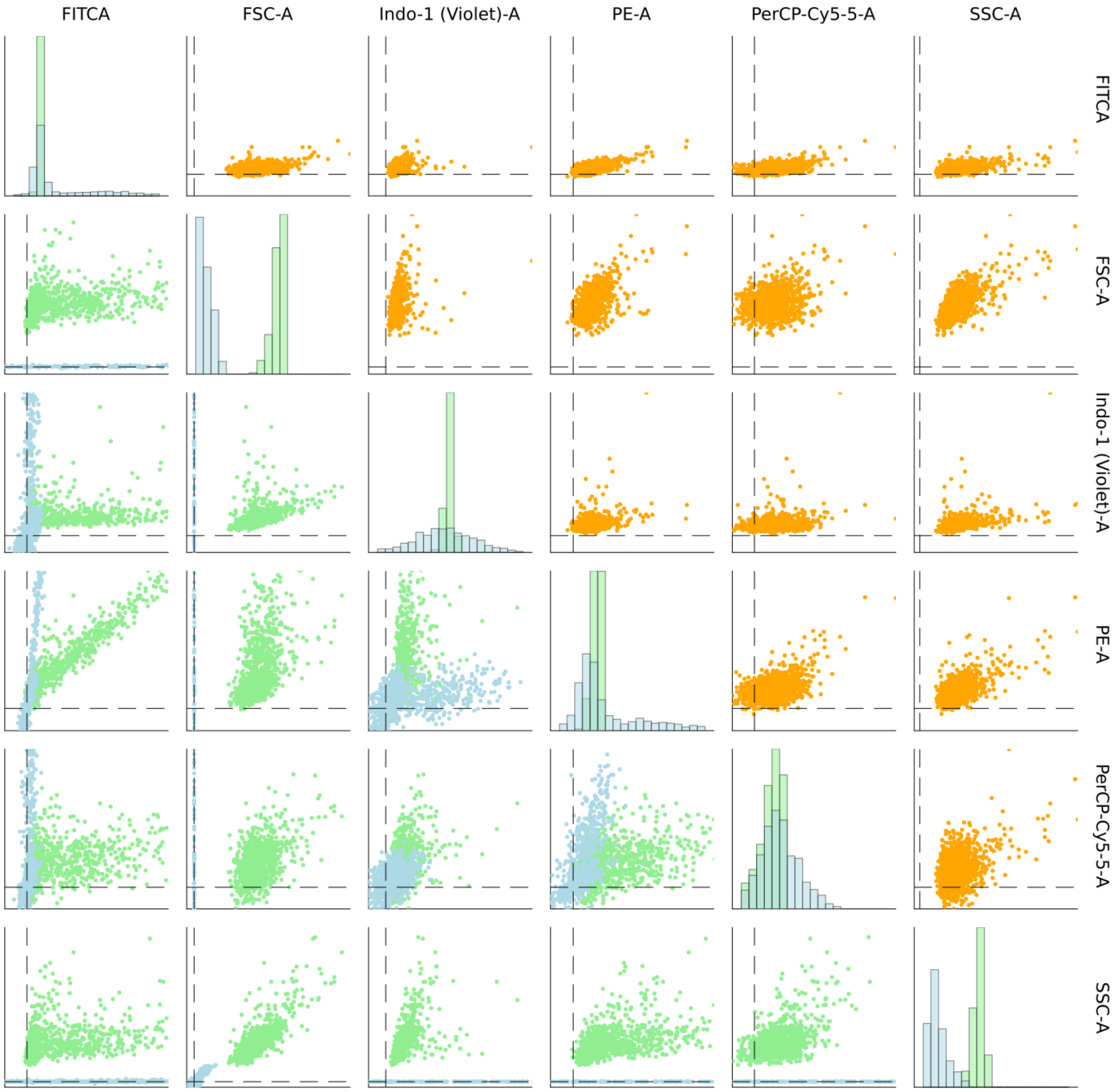

Figure S5: **Scatter plot sections of the multichannel dataset, Related to Figure 5.** The top right triangle shows the autofluorescence distribution (orange). The low left triangle shows the convolution data (light green) and the deconvolution (light blue). In the diagonal, we show the 1D distributions of the convolved and deconvolved results. The horizontal and vertical dashed lines in the off-diagonal plots represent the zero axes in each case.
